# Methods of Liposomes Preparation: Formation and Control Factors of Versatile Nanocarriers for Biomedical and Nanomedicine Application

**DOI:** 10.3390/pharmaceutics14030543

**Published:** 2022-02-28

**Authors:** Domenico Lombardo, Mikhail A. Kiselev

**Affiliations:** 1 Consiglio Nazionale delle Ricerche, Istituto per i Processi Chimico-Fisici, 98158 Messina, Italy; 2 Frank Laboratory of Neutron Physics, Joint Institute for Nuclear Research, 141980 Dubna, Moscow Region, Russia; kiselev@jinr.ru; 3 Department of Nuclear Physics, Dubna State University, 141980 Dubna, Moscow Region, Russia; 4 Physics Department, Lomonosov Moscow State University, 119991 Moscow, Moscow Region, Russia

**Keywords:** liposome formation, lipid-based nanocarriers, phospholipids self-assembly, drug delivery, nanomedicine

## Abstract

Liposomes are nano-sized spherical vesicles composed of an aqueous core surrounded by one (or more) phospholipid bilayer shells. Owing to their high biocompatibility, chemical composition variability, and ease of preparation, as well as their large variety of structural properties, liposomes have been employed in a large variety of nanomedicine and biomedical applications, including nanocarriers for drug delivery, in nutraceutical fields, for immunoassays, clinical diagnostics, tissue engineering, and theranostics formulations. Particularly important is the role of liposomes in drug-delivery applications, as they improve the performance of the encapsulated drugs, reducing side effects and toxicity by enhancing its in vitro- and in vivo-controlled delivery and activity. These applications stimulated a great effort for the scale-up of the formation processes in view of suitable industrial development. Despite the improvements of conventional approaches and the development of novel routes of liposome preparation, their intrinsic sensitivity to mechanical and chemical actions is responsible for some critical issues connected with a limited colloidal stability and reduced entrapment efficiency of cargo molecules. This article analyzes the main features of the formation and fabrication techniques of liposome nanocarriers, with a special focus on the structure, parameters, and the critical factors that influence the development of a suitable and stable formulation. Recent developments and new methods for liposome preparation are also discussed, with the objective of updating the reader and providing future directions for research and development.

## 1. Introduction

Liposomes represent versatile nanoplatforms for the improved delivery of pharmaceutical drugs and active compounds in a large variety of biomedical and nanomedicine applications [1,2]. They are characterized by easily controllable properties such as lipid composition, size, structure and morphology, surface charge, and the possibility of functionalizing their surfaces with polymers or ligands [3,4,5]. Particularly interesting is the ability of liposomal systems to encapsulate both hydrophilic and lipophilic active compounds as well as various biomolecules, including carbohydrates [6], proteins and peptides [7], DNA [8], or imaging compounds [9]. Liposomes’ structure is regulated by soft interactions and self-assembly phenomena that regulate their structural properties and their stability within the environments of biological tissues [10,11,12]. The inclusion of drugs within the vesicles’ nanostructure favors the active compounds’ solubilization in solution and protects against their chemical and biological degradation. The use of liposome nanoformulations also causes a sensitive enhancement of their therapeutic performances [12,13,14,15]. Particularly interesting is the development of new liposome nano-platforms for biomedical and nanomedicine applications, which are stimulated by the liposomes’ special properties, such as their colloidal stability, efficient targeting, and site-specific delivery via various routes of administration [1,2,3,4].

The industrial applications of liposome nanoplatforms include their use as drug-delivery vehicles in nanomedicine, cancer, antimicrobial therapy, as signal carriers in biomedical diagnostics and biochemistry, as adjuvants in vaccination, and as solubilizers and support matrices for various active compounds and macromolecules [13,14,15]. Moreover, owing to their high biocompatibility and non-toxicity, liposomes are the most important category of clinically approved therapeutic drug nanocarriers for cancer treatment [16,17,18]. Those systems play a crucial role also for the encapsulation of unstable bioactive substances (including antioxidants, antimicrobials, phytochemicals, and nutraceuticals) due to their strong enhancement of the colloidal stability [19,20,21].

The modern generation of liposomes includes lipid-based targeted and theranostic nanoplatforms, obtained by the engineering of the phospholipid nanostructures [22,23,24,25,26]. All those varieties of liposome nanoplatforms stimulated a great effort for the scale-up of the fabrication methods in view of industrial developments. Concerning the manufacturing methods, the main critical issues are the low colloidal stability, low entrapment efficiency, toxicity of organic solvents residue, and high cost for large-scale production. Despite the large success in nanomedicine applications, a number of critical issues have been identified which are mainly connected with the poor colloidal stability in biological environments, caused by lipids’ hydrolysis and oxidation processes, particle fission and fusion, and the consequent loss of their active cargo.

In this article, we discuss the main features of the formation and fabrication techniques of liposome nanocarriers with a special focus on the structural properties as well as the crucial factors that influence the development of suitable and stable formulations. We also describe the main positive (and negative) aspects of each approach, as well as their potential for large-scale industrial production.

## 2. Structural Features and Main Control Factors of Liposomes

Liposomes are composed of a spherical hollow structure formed by phospholipids dispersed in aqueous solution. The liposomes’ final organization, structure, and physico-chemical properties depends on the types, size, morphology, concentration, and charge of the constituent lipids, as well as the solution properties (such as the ionic strength, pH, temperature) [27,28]. According to the theory of the lipid bilayers elasticity proposed by Helfrich [29], the curvature energy of a vesicle bilayer is higher than in the (stacked) multilamellar (liquid-crystal) phase (in water excess). Therefore, an energy cost is requested in order to stimulate the curvature of the lipids’ bilayer into a vesicle structure (i.e., a closed lipid bilayer). Therefore, liposomes are metastable nanostructured systems that depend on the methods of preparation (i.e., stirring, sonication, evaporation, extrusion) [29,30].

A crucial parameter for preparing liposomes by a self-assembly process is the critical micelles concentration (CMC), whereby the amphiphilic solution exhibits sensitive changes in their physico-chemical properties [31,32,33], while the aggregation of the amphiphilic lipid molecules produce micelle-like aggregates. The CMC value generally depends on several parameters, such as the hydrophobic/hydrophilic balance of the component lipids, the temperature, and the solvent’s composition and properties (ionic strength, pH) [31,32]. Other important factors of liposome nanocarriers are their size and lamellarity.

**Figure 1:**
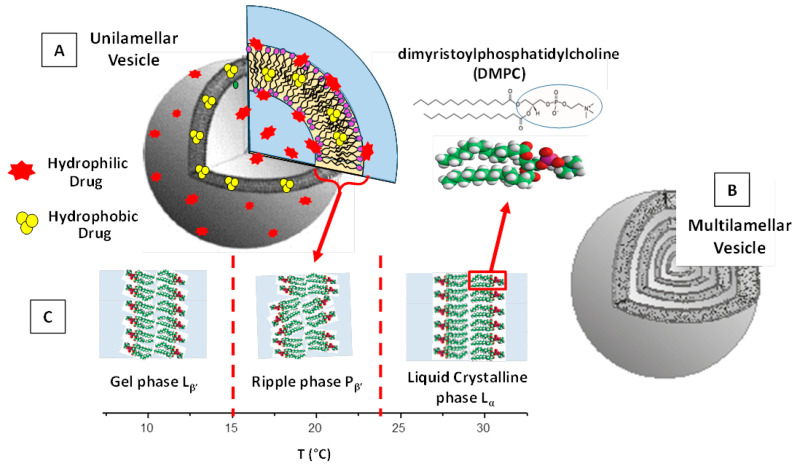
Schematic representation a DMPC unilamellar liposome (**A**). Typical onion-like structure composed of concentric bilayer surfaces (hydrated multilayers) of a multilamellar vesicle (MLV) (**B**). Characteristic phases of a water solution of DMPC phospholipids (**C**).

The size of liposome nanoformulations (that, for biomedical applications, ranges preferably between 50 and 500 nm) strongly influence their drug-delivery process [34,35]. Liposomes with diameters in the range of 100–150 nm favor the cell uptake and are able to escape from the blood vessels’ capillaries within the diseased tissues (such as kidney, heart, lung) and enter through the (fenestrated) vessels into the tumor environments [35,36]. Moreover, liposomes within 50–100 nm (or less) in size are able to avoid immune system phagocytosis clearance and exhibit longer blood circulation times [35,36]. The main structural features of liposomes are reported in Figure 1.

Concerning the lamellarity of the underlying vesicle structures, liposomes may be classified into small unilamellar vesicles < 100 nm (SUVs), large unilamellar vesicles 100–1000 nm (LUVs), and giant unilamellar vesicles > 1 μm (GUVs). Finally, the multilamellar vesicles (MLVs) present an onion-like structure composed of concentric bilayer surfaces (hydrated multilayers) (Figure 1B). ULVs present a more rapid drug-release rate than MLVs, which, on the other side, exhibit a larger entrapped volume.

Another important property of liposomes is given by the fluidity (and rigidity) of their lipid bilayer structure. This property facilitates the crossing of the bilayers by the drug (macro-) molecules and strongly influences the rate of the drug-release process. Owing to their high flexibility, the self-assembled bilayers’ structures undergo a large variety of structural and dynamic transitions that depend on various parameters such as the lipids’ temperature and composition [37,38,39,40]. With increasing temperature. several lipid bilayers pass from an ordered, crystalline (or gel) phase to a fluid state. For example, in Figure 1C, we report the structural feature of dimyristoylphosphatidylcholine (DMPC) MLVs in water solution at different temperature intervals as revealed by small-angle SANS and SAXS experiments [39,40]. This liposome system undergoes structural transitions at the so called “pre-transition” (at temperature T_p_ = 15.0 °C) and the main phase transition (at the temperature T_m_ = 23.4 °C), which identifies the border between the gel L_β’_, ripple P_β’_, and liquid-crystalline L_α_ phases, respectively (Figure 1C).

The presence of an intermediate ripple phase (formed by domains of liquid-crystalline ordered phases within the gel phase) depends mainly on the liposomes’ aggregation state, and is directly related to the phospholipid composition and temperature. The characteristic temperature (T_C_) at which phospholipids undergo the transition from the gel to the liquid-crystalline phase is an important parameter in the formation of liposomes, as it is indicative of liposomes’ fluidity (and permeability) and depends on the alkyl chains’ lengths, their saturation degree, head group species, and the associated charge [27,28]. For T < T_C_, the lipid bilayers are in the gel phase and exhibit lower fluidity (and lower permeability), while for T > T_C_, they are in a liquid-crystalline state and have a larger fluidity (and larger permeability) [37,38,39,40]. Therefore, the phase transition behaviour of the constituent lipids can be exploited to improve liposome structural modification (or integrity). With this aim, a proper lipid composition can be designed to preserve the liposome structure characteristics and their physico-chemical properties, or to stimulate a structural modification (such as an aggregation or drug release) close to body temperature (T = 37 °C).

During the transition from the (more ordered) gel phase to the (less ordered) liquid-crystalline phase, drug molecules are less impeded when crossing the lipids’ bilayer, exhibiting an increase of the permeation rate (with a peak near T_m_). For this reason, liposomes’ nanoformulations, containing lipids with high T_m_, such as the saturated-phospholipids dipalmitoyl phosphatidylcholine (DPPC) or the fully saturated distearoylphosphatidylcholine (DSPC), exhibit a more rigid and stable bilayer structure and a reduced leakage of the encapsulated drugs (weak permeability) [41,42,43]. On the contrary, liposomes containing unsaturated phospholipids (such as the egg or soybean phosphatidylcholine) provide less-stable bilayer nanostructures, caused by the disruption of the packing effect of adjacent acyl chains, and exhibit higher flexibility (and a higher permeability) of the whole lipid bilayer, and then a decrease of T_m_ [41,42,43]. The proper combination of lipids with acyl chains of different types favors, then, the design of temperature-sensitive liposomes (with required T_m_).

Another important factor of the self-assembled lipids’ nanostructures is given by the critical packing parameter CPP = V_0_/A_0_lc (where V_0_ is the effective volume occupied by hydrophobic chains, A_0_ is the effective hydrophilic headgroup surface area, and l_c_ is the maximum effective chain length) [31,44]. The CPP of a certain lipid (or lipids’ composition) allows for predicting the preferred lipid aggregates’ structure, which can be spherical or ellipsoidal (CPP ≤ 1/3), cylindrical (CPP ≤ 1/2), or lamellar (CPP = 1). For (1/2 ≤ CPP ≤ 1), vesicles are generally generated.

The suitable combination of phospholipids with different CPPs or the modulation of the lipid/cholesterol ratio allows for obtaining the optimum size of liposomes. For example, the lipids 1-palmitoyl-2-oleoyl-sn-glycero-3-phosphocholine (POPC), which have CPPs near to 1, are able to form liposomes. Most of the naturally occurring phosphatidylcholines form planar bilayers (CPP = 1), but when mixed with conically shaped phospholipids that favor the bilayer curvature, (e.g., 1,2-dioleoyl-sn-glycero-3-phosphoethanolamine (DOPE)), liposome nanostructures are also formed [43,44,45]. It is worth pointing out that, for amphiphilic building blocks with a more complex geometry or in the presence of a complex combination of (short- or long-range) interactions, the size and shape of amphiphilic nanoassemblies is hard to predict with high precision. However, the analysis of the CPP still remains a valid approach for a qualitative estimation of the main morphological features of soft-interacting macromolecules, and a versatile tool for the design of nanoscale drug carriers [45,46,47,48,49].

An important lipid that can sensitively influence the structural properties of liposomes is cholesterol. When incorporated into liposomes, cholesterol decreases the lipid bilayer packing defects by distributing itself with its hydroxyl group close to the head lipids’ group region, while the aromatic rings are aligned with the hydrophobic alkyl chains. This configuration causes an increased fluidity in the bilayer core, but an increase of the viscosity (and rigidity) in the headgroups’ region. This causes a decrease of the fluidity and water permeability of liposomes, while the bilayer is less inclined to penetration (absorption) by external nanoparticles. This increase in the mechanical rigidity results is important for the liposome stability and prevents its interaction with proteins (such as transferrin, albumin, and high-density lipoproteins), thus avoiding a possible reduction of their performances [34,35]. Moreover, the ability of the cholesterol to control the phospholipid packing, membrane fluidity, and the liposomes’ surface charge, produces an effect also on the liposome size, final morphology, and encapsulation efficiency [50]. Due to its low, flexible, hydrophobic ring structure, cholesterol can interact (through hydrophobic interactions and cooperative hydrogen bonds) with the phospholipid hydrophobic acyl chains, while its presence stabilizes the straight-chain arrangement of saturated fatty acids (through the van der Waals interactions) [50,51]. Recent results evidenced that the inclusion of cholesterol in liposomes causes a sensitive increase of the incorporation efficiency of retinol, as well as an increase of the mean size and the colloidal stability of the liposome nanocarriers [51]. Recently, cholesterol proved to be a crucial component in modulating the release of encapsulated hydrophilic (fluorescent dye) sulforhodamine B (SRB) molecules. The increase of cholesterol concentration induced a decrease in the bilayer fluidity and an increase in the mean liposome size, (with a transition from irregular to regular spherical-shape vesicles) [52].

One approach to enhance the colloidal stability of liposomes consists of the incorporation of charged components (such as anionic/cationic lipids or charged macromolecules). The charged surface creates, in fact, an electrostatic repulsion (a so-called ζ-potential) among liposomes that prevents possible coagulation (or aggregation) effects. Several investigations evidenced that a negative charge (induced by the inclusion of negatively charged macromolecules or nanoparticles) can provide an enhancement of the colloidal stability of neutral liposomes, due to the generated electrostatic repulsive forces [53,54,55,56,57,58]. Moreover, negatively charged lipids (such as DOPS and DMPG) are recognized by macrophages (by an aggregation-dependent phagocytic uptake mechanism) and are able to enter the cells via endocytosis (with a faster rate than the neutral lipids), thus resulting in a shorter circulation time. Within the blood circulation, the liposomes interact with the biological fluids and undergo an opsonization process with the circulating proteins, followed by the uptake by the MPS. [59]. On the contrary, for gene delivery, the cationic liposomes are generally preferred, as in the case of the charge interaction between positively charged lipids, such as dioleoylphosphatidylethanolamine (DOPE), which have an amine head group (NH_3_^+^), and the nucleic acids (negatively charged) [57]. Cationic liposome nanocarriers favor the interactions with glycoproteins, which are present on the endothelial cells’ membranes, thus exhibiting longer circulation half-lives [57,58]. However, cationic liposomes may interact with the anionic components of the blood (such as plasma proteins), and may induce an enhanced uptake by the mononuclear phagocytic system (MPS), thus favoring the clearance process by the liver, lung, or spleen [59]. This causes a diminished accumulation in tumor tissues.

The enhancement of the liposomes’ colloidal stability can be obtained through the incorporation of specific polymers into their surface that hinder (sterically) the components of the blood from the interaction with the surface of the liposomes. This effect can be obtained through the liposome PEGylation process, which consists in the liposome surface functionalization with polyethylene glycol (PEG), thus improving the colloidal stability and the blood-circulation time of active therapeutics [60,61]. However, a number of investigations have reported adverse immune responses, such the accelerated blood clearance (ABC), consisting in the rapid clearance of PEGylated nanocarriers upon repeat administration [62,63], or the complement activation-related pseudo-allergy (CARPA), consisting in an adverse reactivity (hypersensitivity), which is correlated with side effects caused by the action of the PEGylated nanocarriers [63]. These (adverse) immune responses stimulate the investigation of alternative (natural or synthetic) polymers that are able to propose the same properties and functions of PEG.

Finally, it is possible to functionalize the surface of the liposomes with a large variety of ligands (including monoclonal antibodies, peptides, aptamers, and growth factors) the favors the specificity of the liposome interaction (targeted drug delivery) and the controlled drug release to specific target sites (such as diseased tissues or tumors) [1,64]

**Figure 2:**
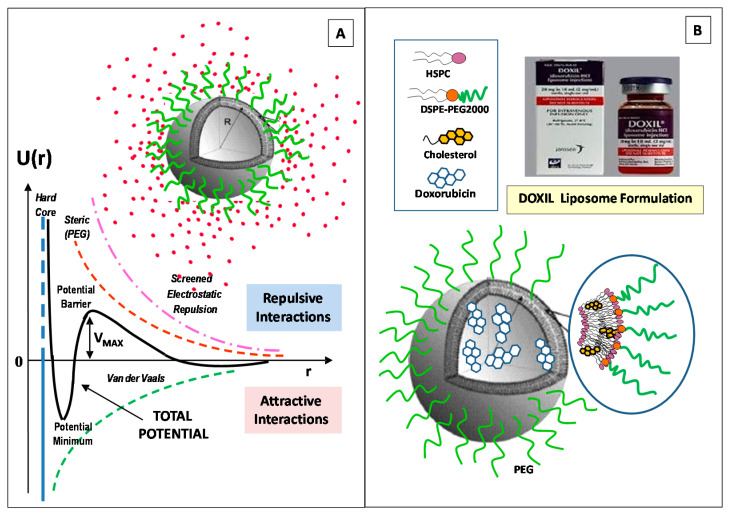
Schematic representation of the main interactions exhibited by liposomes (**A**). Main structural characteristics of the anticancer drug Doxil (**B**).

In Figure 2A, we report the main interactions exhibited by liposomes. We also report the main features of the (first FDA-approved) Doxil anticancer nanoformulation, a PEGylated liposome doxorubicin nanoformulation, for the treatment of epithelial ovarian Kaposi’s sarcoma [16,17,18]. The energy barrier which results from the balance between attractive and repulsive forces prevents the aggregation (adhesion) of two nanocarriers while approaching one another. The control over the nanocarriers’ soft interactions represents, then, a fundamental step for the design and engineering of the colloidal stability and biocompatibility of the liposomes, in view of overcoming the number of obstacles and biological barriers found in biological system [3,58].

In summary, the design and engineering of all the factors and parameters mentioned above, such as the lipid chemical nature and headgroup charge, the length and degree of unsaturation of the alkyl hydrophobic chains, the transition temperature (T_c_), as well as the liposome surface functionalization (with PEG, ligands, proteins, or antibodies), make liposomes versatile tools in a large variety of biomedical applications.

## 3. Conventional Methods for the Preparation of Liposomes

The main goals of a method for liposome nanoformulation formation is the formation of monodisperse particles (with a narrow size distribution) and the requested degree of lamellarity, efficient drug inclusion, and long-term colloidal stability of the products. In the conventional methods, liposomes, initially dissolved in a volatile organic solvent, are subsequently mixed with an aqueous phase. The presence of an organic solvent may perturbate the chemical properties of the incorporated active compounds, or influence the stability (or toxicity) of the generated nanoformulation [65]. The conventional methods for liposomes preparation involve the following main stages:

1. Dissolution of lipids in an organic solvent;
2. Drying-down of the resultant lipidic solution from the organic solvent;
3. Hydrating the lipid with an aqueous media (followed by agitation/stirring);
4. Downsizing (and/or change in lamellarity);
5. Post-formation processing (purification, sterilization);
6. Characterization of the final nanoformulation product.

Depending on the specific formation process, the hydration of the lipid (stage 3) may anticipate the dry-down of the lipid solution from the organic solvent (stage 2).

### 3.1. Thin-Film Hydration (TFH) Method (Bangham Method)

**Figure 3:**
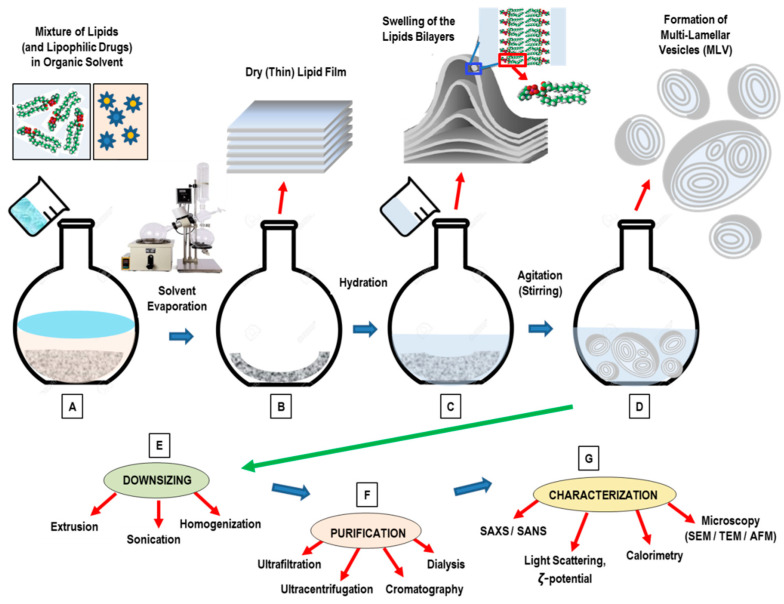
Schematic representation of the main stages of the thin-film hydration method of liposome preparation. The main lipid components (and eventually lipophilic drugs/macromolecules) are dissolved in organic solvent (**A**). After the evaporation of the solvent, a dry (thin) lipid film is formed (**B**). The lipid film is then rehydrated in a saline buffer (eventually containing hydrophilic dugs to be entrapped), causing a swelling of the lipid bilayers’ stacks (**C**). The successive agitation/stirring of the sample favors the formation of (polydispersed) multilamellar vesicles (**D**). The final stages of the production process include the liposomes’ downsizing (**E**), purification (**F**), and characterization (**G**).

The thin-film hydration technique (the so-called Bangham method) is the oldest, most common, and simplest method used for the preparation of MLVs [66,67,68] (Figure 3). To ensure a homogeneous mixture, the main phospholipid ingredients are dissolved in an organic solvent (such as dichloromethane, chloroform, ethanol, or a chloroform–methanol mixture) (Figure 3A). Successively, the evaporation under vacuum pump at a temperature of 45–60 °C allows for the removal of the organic solvent. For small volumes (<1 mL), the organic solvent may be evaporated by means of a dry nitrogen or argon stream in a fume hood until the residual organic solvent is completely removed, while a rotary evaporation is usually used for larger volumes. After the removal of the organic solvent, a homogeneous, dry, thin-lipid film (of stacked bilayers) is then formed (Figure 3B). The final stage consists in the hydration of the lipid film (Figure 3C) using an appropriate aqueous medium (buffer) that, for the pharmaceutical formulation, may consist of a solution of simple distilled water, or a normal (phosphate) saline buffer at pH 7.4 [65,66,67,68]. The hydration process (with duration 1–2 h) is generally performed at a temperature of 60–70 °C, and in any case, above the phase-transition temperature of the component lipids. During this stage, the agitation (stirring) may help to detach the (swelling) lipids’ lamellae from the internal vessel surface. Facilitating the full lipid hydration, the final liposome suspension is then left overnight at a temperature of T = 4 °C. During the hydration stage, the lipid becomes swollen and hydrated, resulting in the formation of a MLV suspension that is highly heterogeneous in size and lamellarity (Figure 3D).

Concerning the drug loading into liposomes systems, lipophilic drugs can be dissolved with the phospholipids mixture prior to the thin film formation, while hydrophilic cargoes can be inserted within the hydration mediums and then incorporated (passively) into the liposome during the hydration process. The successive step of the formation process consists in the reduction of the liposomes’ size and lamellarity. The main drawbacks of the Bangham method are the difficulty of removing the organic solvent, the low entrapment efficiency, and the small-scale production.

By means of the TFH method, liposome nanoformulations have been employed to encapsulate a large variety of lipophilic drug molecules, such as Docetaxel (DTX), Paclitaxel (PTX), Quercetin, Resveratrol (RES), as well a variety of hydrophilic ingredients (such as targeted protein, small interfering RNA, siRNA) [66,67,68]. Recently, Jeon et al. [69] developed a theranostic multilayered nanomaterial by inserting an additional liposomal layer (LAL) to the gold (Au)-coated liposome prepared by a TFH method. The additional liposomal layer enabled further functionalization with PEG groups (to enhance in vivo stability) and radiolabeling (for in vivo imaging). In vivo photothermal therapy (PTT) investigations evidenced that the suitable combination of intravenous injection of LAL and laser irradiation were able to suppress the tumor progression in 4T1 orthotopic tumor mouse model [69]. This liposomal nanocarrier could be a promising theranostic PTT nanoplatform for the treatment of metastatic lesions, as it exhibited high stability, tumor targeting efficiency, and imaging ability. Wang et al. [70], developed a (pH-responsive) betulinic acid-loaded liposome (pH-BA-LP), coated with Eudragit S100 by means of the TFH method and the (easily scalable) pH-driven method. The prepared liposomes showed advantages such as large encapsulation efficiency (of 90%), low size (<100 nm), and high stability. Concerning the in vivo antitumor functions, it was shown that the tumor proliferation and cell migration were significantly inhibited in colorectal cancer after the action of the pH-BA-LP nanocarriers. The pH-BA-LP also inhibited tumor growth, with potential antitumor effects connected with the enhancement of the autoimmunity level in tumor-bearing mice. This study evidences that TFH method still represents an effective technique for the development of pH-responsive liposome nanoformulations for the delivery of biologically active drugs, with potential improvements of the therapeutic index in chemotherapy treatments [70].

### 3.2. Detergent Removal (Depletion) Method

With the detergent removal method, lipids are hydrated (and solubilized) by using a detergents solution [71]. Upon mixing, the detergent will associate with the phospholipids (shielding the hydrophobic portions from the direct interaction with the aqueous phase), and thus, mixed (detergent/lipids) micelles are formed. With the successive (progressive) removal of the detergent, the mixed micelles become richer in lipids and give rise to the formation of unilamellar vesicles [65,72]. Commonly used detergents are those with a high CMC, such as sodium cholate, Triton X-100, sodium deoxycholate, and alkyl glycoside [65,72]. Detergent removal can be obtained through different routes.

The simplest method for detergent removal is the dilution method (by 10- to 100-fold) by means of a buffer. Upon dilution with a buffer of the aqueous solution of a mixed lipid–detergent system, the size and polydispersity of the initial micelles increases [30]. Finally, a spontaneous transition from polydispersed (elongated) micelles to vesicles occurs, as the system is diluted beyond the mixed micellar phase boundary [30]. In the aqueous solution of a mixed system composed of lecithin-bile salt (detergent), with the increase of the dilution factor, the aggregates progressively passed from spherical micelles, to longer (flexible) cylindrical micelles, until becoming nearly monodisperse unilamellar vesicles (at the higher dilution factors) [73]. This sequence can be explained on the basis of the concept of spontaneous curvature (and the critical packing factor). While lecithin alone forms aggregates of low spontaneous curvatures, bile salt alone forms highly curved (spherical) micelles. At high bile salt contents, therefore, spherical (or elongated) mixed micelles are formed within the mixed lecithin-bile salt system. Because bile salt is far more water-soluble than lecithin, a subsequent dilution causes a reduction of the bile salt content within the aggregates, and this causes a decrease of the spontaneous monolayer curvature (which leads to the formation of liposomes) [73].

In conclusion, in the final stage of the detergent removal method, when the total detergent concentration becomes lower than the detergent’s CMC, (proteo-) liposomes will form, while other methods should be used to remove the residual detergent remaining in the nanoformulation. The detergent removal method has the main drawbacks of a final low concentration of liposomes, and a low entrapment efficiency of hydrophobic compounds.

An alternative approach for detergent removal is the detergent dialysis method, which furnishes an excellent reproducibility, with the final formation of homogenous size populations of liposomes. However, with this approach, traces of detergent(s) are still present within the liposomal nanoformulation. Finally, column gel chromatography, centrifugation, and the adsorption onto hydrophobic resin beads have been used as alternative efficient approaches for detergent removal [65,72].

**Figure 4:**
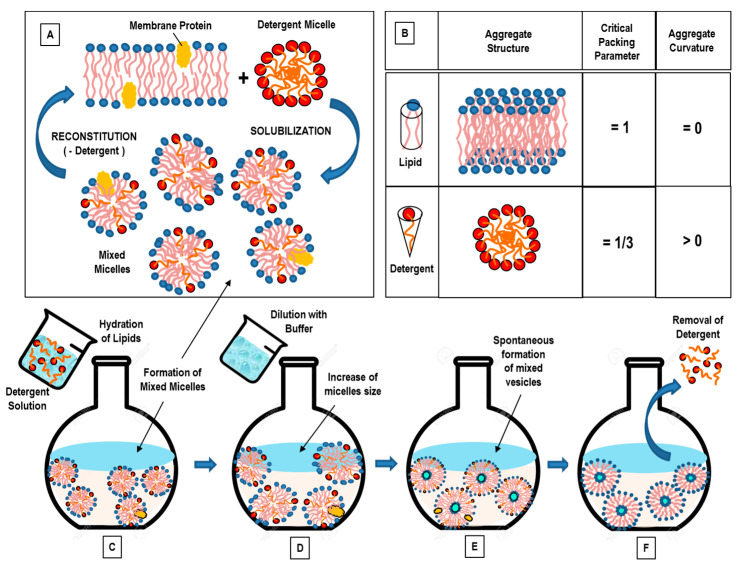
Self-assembly process in mixtures of lipids and detergents. (**A**) Membrane solubilization and reconstitution by addition (or removal) of detergents. (**B**) Characteristic molecular geometries’ and aggregates’ structures of (pure) lipids and detergents. (**C**–**F**) Main stages of the detergent removal method. Initially, the lipid hydration with a detergent solution allows for the formation of mixed (detergent/lipids) micelles (**C**). The successive dilution of mixed micellar solution with aqueous buffer favors an increase of the mixed micelles’ size (and polydispersity) (**D**), followed by a transition to the vesicles’ structures (**E**). The formation process is completed by a complementary method for the removal of the residual detergent inside the liposomal nanoformulation (**F**).

The self-assembly process which underlies the detergent removal method is driven by the molecular structure of the involved amphiphiles (Figure 4A). Most of phospholipids have a cylindrical molecular conformation (and then a critical packing parameter of CPP = 1), and in aqueous solution, they likely form bilayers (curvature = 0). On the contrary, most detergents have a cone structure (with CPP = 1/3), and favor the formation of micellar aggregates in solution (Figure 4B). When lipid bilayers (which can include proteins) interact with a (micellar) detergent solution, lipid–protein–detergent mixed micelles are formed (solubilization). The formation process can be reversed by removing the detergent (reconstitution) (Figure 4A). The characteristic molecular geometries’ and aggregates’ structures of (pure) lipids and detergents are also reported (Figure 4B). In Figure 4C–F, we report the main stages of the detergent removal method.

The detergent removal technique permits the vesicles’ formation with no degradation of their relevant biological activity, and represents one of most employed methods for the reconstitution of (poorly soluble) membrane proteins [65,71].

Different studies have investigated the micelle-to-vesicle transition (MVT) process, by which mixed micelles transform into vesicles, and their effect on the reconstituted (or encapsulated) components onto liposomes, by describing the (molecular and supramolecular) out-of-equilibrium processes and providing quantitative information on the intermediate (unstable) aggregates, partition coefficients, etc. [72,73,74,75,76,77].

Recently, proteoliposomes (complex composed by integral membrane proteins (IMPs) inserted within unilamellar liposomes) have been employed as model systems to investigate the structure/function relationships between proteins and biological membranes [78]. Proteoliposomes are formed by removing the detergent from solubilized lipid/membrane protein mixtures or from mixtures of detergent-solubilized membrane proteins and preformed liposomes [78]. Proteoliposomes mimic isolated cells, while the specific bio-environments of compounds (such as ions, or pH gradients) can be created inside (and/or outside) the liposome system. This approach favors in vitro biomembrane studies, and provides important information about the integral membrane proteins (IMPs) structure–function relationship, thus stimulating pharmaceutical developments concerning the protein activity. Different liposome nanoplatforms (prepared by mixing anionic and conical lipids) were developed to investigate the activity of mammalian glucose transporters and the correlated IMPs’ functional conformations [78]. Recently, Neves et al. [79] reported the reconstitution of OmpF in preformed DMPC and E. coli liposomes using two different techniques for detergent removal: (1) exclusion chromatography and (2) incubation with detergent-adsorbing beads. The study evidenced that protein insertion in membranes strongly depends both on the lipid composition used for the liposomes’ formation and the approach used for the detergent removal. Despite the extensive investigations and diverse applications of the reconstitution process, the mechanism of liposome reconstitution (i.e., insertion process of the membrane proteins into liposomes) is still not fully understood.

Finally, the main advantages of the detergent removal method are the good control over the particle dimension and the product homogeneity, which strongly depend on the detergent removal rate and the initial detergent/phospholipid ratio. Some potential disadvantages of this method are connected with the slow equilibration process of the intermediate micellar aggregates, the presence of detergent residues, and the difficulty of removing the organic solvent.

### 3.3. Solvent Injection Method

The solvent injection methods consist in the lipid dissolution into an organic solvent, and the injection of the solution into aqueous phase. Two main solvents (ethanol and ether) have been employed for the preparation of liposomal nanoformulation [65,66,67,68].

#### 3.3.1. Ethanol Injection Method

**Figure 5:**
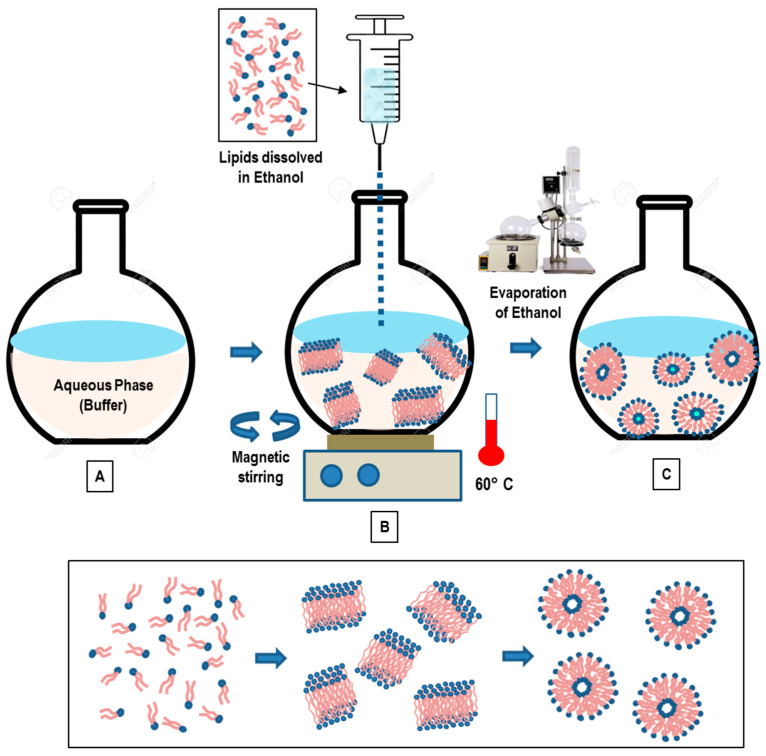
Schematic representation of the main stages of the ethanol injection method. A composition of lipids dissolved in alcohol solution is injected into an aqueous phase (buffer) (**A**). The dilution of ethanol in the water solution favors the self-assembly of lipid components and the formation of bilayer planar fragments (**B**). Finally, the ethanol evaporation (depletion) favors the fusion of the lipids’ fragments and the formation of closed unilamellar vesicles (SUL and LUV) (**C**).

In the ethanol injection method, the phospholipids (dissolved in ethanol) are rapidly injected to a (pre-heated) distilled water (or TRIS-HCl) buffer. The dilution of ethanol in the water solution below a critical concentration favors the self-assembly of the dissolved lipids in the aqueous phase [80,81]. The rapid ethanol dilution (in the aqueous phase) also favors the lipid molecules’ precipitation and the successive formation of bilayer planar fragments (stacks), which encapsulate the aqueous phase. Finally, the ethanol depletion (evaporation) favors the fusion of the lipids’ fragments and the successive formation of closed unilamellar vesicles. In Figure 5, a schematic representation of the main stages of the ethanol injection method is reported.

The volume of added ethanol represents a crucial factor of the liposome formation. If the ethanol does not exceed 7.5% of the whole formulation volume, homogenous SUVs are formed. Conversely, if ethanol is rapidly injected (to a huge excess of buffer) a heterogeneous population of MLVs are formed. [81]. The residual ethanol is separated by a dialysis membrane, while the use of a filtration tube (under the pressure of nitrogen gas) allows for obtaining the concentration of the sample [67]. With this method, both LUV and SUV liposomes are spontaneously formed. Finally, the ethanol can be removed by using a rotary evaporator (under nitrogen gas at reduced pressure, and T = 40 °C) [81].

Recently, an automated high-throughput version of the ethanol injection method has been developed, which uses a dedicated pipetting robot (for measuring and mixing volumes, mixing reservoir) in connection with a dynamic light scattering plate reader to characterize the liposomes in terms of size/distribution. This automated version favors the optimization of the amount of used materials, decreases the liposomes’ production time (and costs), and facilitates the screening of many liposome properties in a shortened time [81].

The ethanol injection method was employed for the encapsulation into liposomes of the hydrophobic beclomethasone dipropionate (BDP) and hydrophilic cytarabine (Ara-C) drugs, with the aim of realizing an efficient nanocarrier to be administered via the pulmonary route [82]. The drug-loaded liposomes were characterized in terms of size, encapsulation efficiency (EE), release study, cell uptake, and aerodynamic behavior, as a function of the main formulation parameters. The results evidenced the formation of small multilamellar vesicles, with sizes ranging from about 80 to 170 nm, and with an higher encapsulation efficiency of about 100% for the hydrophobic BDP drug, and about 16% for the hydrophilic (Ara-C) drugs. The in vitro release study showed a prolonged release profile for BDP, in contrast with Ara-C, which was released more rapidly. The cell-uptake experiments evidenced that the (fluorescent) liposomes have been well internalized into the cytoplasm of SW-1573 human lung carcinoma cells, thus confirming the possibility of using liposomes for lung cell targeting. Finally, the nebulized Ara-C and BDP liposomes presented aerodynamic diameters compatible with deep lung deposition, thus confirming that the formed liposomes’ nanoformulation represents an efficient nanocarrier for both Ara-C and BDP pulmonary delivery [82]. Recently, the liposome formulation, consisting of a 1:1 ratio of organic:aqueous phase (*v*/*v*), and the phospholipids DOPE/cholesterol/DSPE-mPEG2000, was used to develop a novel Methotrexate (MTX)-loaded nanocarrier for rheumatoid arthritis therapy by using the ethanol injection method [83]. The study investigated a novel pre-concentration approach, based on the use of an initial aqueous volume of only 20%, and the addition of the remaining 80% after the ethanol evaporation stage. The proposed approach evidenced the formation of small liposomes (130 ± 10 nm) with a small polydispersity index (<0.1), without the need of the successive extrusion process, and a high MTX encapsulation (about 40%). On the contrary, liposome-encapsulated MTX produced by the conventional ethanol injection method exhibited a high value of size (>150 nm) and PDI polydispersity index (>0.1) and were considered not suitable for further in vivo applications, thus requesting a further extrusion process to achieve liposomes suitable for biomedical applications. Moreover, nuclear magnetic resonance studies evidenced the mutual interactions (via hydrogen bonding) between the main phospholipids and the drug, while the in vivo experiments revealed an increased biological benefit in arthritic mice [83]. This approach contributes to a significant advance in rheumatoid arthritis treatments and therapies by using the liposomal nanoformulation of MTX [83].

In conclusion, the main advantages of the ethanol injection technique are the simplicity, the high level of reproducibility, the use of a non-harmful solvent such as ethanol, as well as the easy scale-up of the method. The main drawbacks are connected with the difficulty of removing the residual ethanol (as it forms azeotrope with water), and the final formation of a (very diluted) heterogeneous (30–110 nm) population of liposomes. Finally, there is the risk of an inactivation of (biologically active) macromolecules in the presence of (even low amounts of) ethanol.

#### 3.3.2. Ether Injection Method

In the ether injection approach, lipids dissolved in ether (or diethyl ether/methanol mixture), are (slowly) injected to an aqueous phase containing the components to be encapsulated, which are heated to a temperature range of 55–65 °C (in order to facilitate evaporation of the solvent from the liposomal product). The successive removal of the organic solvent (under reduced pressure) favors the generation of LUVs [84,85]. The injection of an ether solution of lipids into the water phase causes the formation of SUVs from the evaporation of the ether solvent (the so-called ether vaporization method) [84]. An advantage of this approach (compared to the ethanol injection method) consists in the more efficient removal of the organic solvent from the final product. This favors the formation of concentrated liposome solutions with high entrapment efficiencies. The main limits of this method are the high polydispersity of the final population of liposomes (60 to 200 nm) and the fact that the active (or therapeutic) agents are exposed to organic solvents and high temperatures. This circumstance might compromise both the safety and stability of the liposomes’ formulation.

Recently, different liposomes loaded with tamoxifen (a hormone used to treat breast cancer) were prepared by modified ether injection (MEIM) and thin-film hydration methods (TFHM) [86]. The prepared liposomes, characterized by using optical microscopy, evidenced an increased encapsulation efficiency (from about 60% to 86%) as a function of the increasing amount of phospholipids and cholesterol, while in vitro (by means of the dialysis membrane) and ex vivo (by means of the chicken intestinal sac,) diffusion studies evidenced an efficient and controlled release process. The study evidenced a similar performance of the liposome system prepared by the two different methods [86]. A variation of this method is given by the inkjet method, based on the employment of commercial inkjet printers (and cartridges), which are used to inject the lipid solution into the water phase [87]. This approach allows for the formation of highly reproducible SUVs (in the range of 50–200 nm) with high levels of control on particle dimension (with narrow distribution) and efficient drug incorporation within the nanovesicles, as well as a high potential for scaling up [87].

### 3.4. Reverse-Phase Evaporation Method

**Figure 6:**
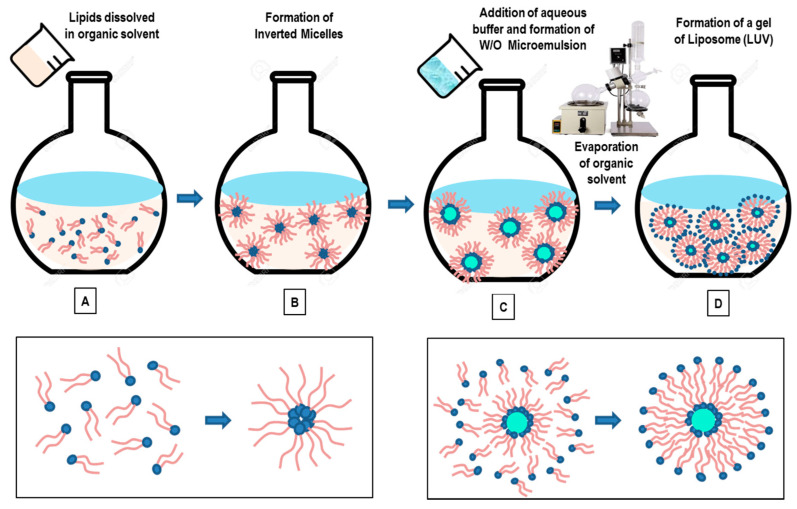
Schematic representation of the main stages of the reverse-phase evaporation method. Lipids are dissolved in organic solvent (**A**), and the formation of inverted micelles is observed (**B**). The addition of aqueous media (buffer), followed by emulsification of the solution, favors the formation a homogeneous dispersion of a W/O microemulsion (**C**). With the final elimination of the organic solvent (by using rotary evaporation, under vacuum), a viscous gel is formed in the solution, which finally collapses to form liposomes (**D**) (LUVs).

In this method, lipids are dissolved in an organic solvent (Figure 6A) (such as a mixture of diethyl ether and chloroform (1:1 *v*/*v*), or diethyl ether/isopropyl ether, or chloroform/methanol (2:1 *v*/*v*)) and favor the formation of inverted micelles (Figure 6B) [88,89,90]. A given quantity of an aqueous phase (buffer) is then added to the solution (Figure 6C). The lipids rearrange themselves at the interface between water and oil, creating a water-in-oil (W/O) microemulsion. The W/O microemulsion can be emulsified, by mechanical or sonication methods, to facilitate the formation of a homogeneous dispersion. With the aim of improving the liposomes’ efficiency, a phosphate saline (or citric-Na2HPO4) buffer is often added to aqueous phase. The use of a continued rotary evaporation (under reduced pressure) allows for the removal of the organic solvent, until the formation of a viscous gel. The slow organic solvent elimination favors the disruption of the inverted micelles and promotes the successive liposomes’ formation (LUVs). At a given critical point, the gel collapses, while the excess of phospholipids in the solution environment distribute themselves around the inverted micelles to form a lipid bilayer around the (residual) water droplets, which results in the liposomes’ formation (Figure 6D) [88,89,90].

The large amount of the aqueous phase encapsulated by the microemulsions favors the encapsulation of a large amount of macromolecules within the liposomes. With this method, it is possible to encapsulate 30–45% of the aqueous volume, while (at optimal conditions) up to 65% of entrapment may be obtained [88]. The main drawbacks of this approach is connected with the presence of residual solvent (which can be removed by means of the dialysis and centrifugation methods) and with the difficulties of scaling-up the process. A further disadvantage of this method is that the process is not suitable for fragile molecules (such as peptides), as the drugs to be loaded within the liposomes are in direct contact with an organic solvent. Finally, biomolecules such as enzymes, proteins, or oligonucleotides may undergo a conformational change due to the mechanical agitation and the direct exposure to the organic solvent (such as protein denaturation, breakage of DNA strands) [88,89,90].

Recently, the reverse-phase evaporation method has been employed to combine therapeutic and diagnostic agents in the same lipid (theranostic) nanoformulation for advanced biomedical applications. Ultra-magnetic liposomes (UMLs), prepared by means of the reverse-phase evaporation method, exhibited a higher magnetic nanoparticle (MNP) loading efficiency (about 100-fold), compared to the classical thin-film hydration method [91]. Do et al. [92] developed nucleic acid-delivery nano-formulation systems based on magnetic cationic liposomes (MCLs), by means of reverse-phase evaporation and cosolvent sonication techniques. The new MCLs’ nano-formulation composed of the lipids DPPC, DSPC, DOPE, 18:0 PEG2000 PE, 14:0 PEG750/1000/2000 PE, and cationic lipid DMAPAP, showed high capacity and efficiency to form complexes and transfect (CT-26) cells (using the antibiotic-free pFAR4-luc plasmid), thanks to their ability to transfect cells with high efficiency. The constructed MCLs (of <200 nm) offer a magnetic resonance imaging contrast enhancement agent (due to the encapsulated magnetic nanoparticles), and can be considered a promising nanovector for image-guided gene-delivery therapy. Recently, a (pressure-controlled) encapsulation of graphene quantum dots (GQDs) into liposome nanocarriers has been obtained by the reverse-phase evaporation method [93]. The GQDs-loaded liposomes exhibited a high loading of ultra-small (~4 nm) GQDs into the aqueous liposomes’ cores (45.68 ± 1.44%), which was controllable by the pressure, and exhibited a very good stability for over a month. Furthermore, the inclusion of the indocyanine green (an near-infrared photothermal agent) could convert NIR laser energy into thermal energy and break down the liposomes, causing the release of GQDs in 6 min. This NIR light-controlled drug-release nanoformulation exhibited a good in vitro (photothermal) therapeutic performance, and 75% of cancer cells were killed at a concentration of 200 μg/mL [93]. The successful development of these controlled-release nanocarriers by the reverse-phase evaporation method may stimulate future biomedical applications of advanced liposome theranostic systems.

## 4. Downsizing and Post-Formation Processing

For specific biomedical applications, the precise control of particle size (and polydispersity index—PDI), lamellarity, and homogeneity, is a crucial step in their manufacturing and a fundamental parameter in the products’ specifications. For this purpose, a post-formation processing is required, with the aim of breaking down the initial large MLVs obtained as the final product. Three main procedures, namely, the sonication, extrusion, and the high-pressure homogenization methods, represent the most employed post-formation treatments of size reduction (downsizing) within the liposome formation approaches.

### 4.1. Sonication Method

The sonication method consists in the application of a high (ultrasonic)-energy input based on cavitation to the MLVs liposome solution under a passive (inert) atmosphere. Two types of sonication techniques are used on an aqueous dispersion of a phospholipid system: namely, the bath sonication and probe sonication techniques [94]. In the probe sonication method (generally used for small volumes), a sonicator tip is immersed into the liposome solution. The bath vessel is immersed into a water/ice bath to avoid high energy delivered by the tip, which causes a local warming-up and degradation of the lipidic solution [65,94]. For this reason, The main disadvantages of this method are connected with the possible release of metal (titanium) particles from probe tip, which may cause contamination of the lipid solution. Moreover, with prolonged sonication times (≥1 h), sensitive amounts of lipids can be de-esterified (≥3%) [65,94]. In the bath sonication method (generally used for large volumes), the liposome dispersion is placed into a sterile vessel (with a temperature-control system), or under an inert atmosphere. The main disadvantages of this approach are the low encapsulation efficacy, possible phospholipid (or encapsulated compound) degradation, and the high size polydispersity [65,94]. Finally, although sonication is one of the most used approaches for the formation of SUVs (with diameters in the range of 15–25 μm), it does not seem optimal in those cases in which precise physical liposome properties are needed.

### 4.2. Extrusion Method

The extrusion method consists in the extrusion through pore-containing membranes (with sizes ranging from 1 mm down to 25 nm). A heating block set around the extruder allows for performing the extrusion above the phase-transition temperature of the phospholipids. Several passes through the polycarbonate membrane filters allow for the formation of (narrow-size distribution) LUV liposomes with dimensions close to the membrane pores’ sizes. This method allows for a reproducible result of the final liposome product, as evidenced by several investigations performed on various lipid formulations [95,96,97]. A variation of this method is given by the maximator device, an extrusion setup consisting of a thermo-stable supply vessel connected to a high-pressure pumping system [96].

The extrusion methods have a high reproducibility of downsizing. The main disadvantage of this method is the sensitive product losses, which represent a limit for large-scale productions. A different extrusion process for the production of liposome nanoformulations, called the French press method, is based on the extrusion, at high pressures, of suspensions of MLV through a small orifice, which results in the formation of SUVs [98]. Liposomes formed with this setup are larger than those obtained by means of the sonication of MLVs [98]. This technique was originally developed to break up cells under more appropriate (milder) conditions than those used with ultrasound methods (to avoid the degradation of lipids, proteins, or other sensitive biomolecules during the sonication process). The drawbacks of the method are connected with the difficulty of reaching high temperatures, and the relatively small working volumes (<50 mL), which are not suitable for large-scale production [98].

### 4.3. High-Pressure Homogenization Method

In the homogenization method, the initial liposome suspension (composed of multilamellar liposomes) is continuously injected with a constant high pressure through an orifice, and collides with a fixed stainless-steel wall that causes downsizing of the liposomes [99]. The formation of the liposomes’ structure takes place due to cavitation, shear phenomena, and turbulence. With this method, the liposome size distribution may still be broad and variable. More specifically, the properties and the size distribution of the liposomes depends on the pressure, temperature, and the number of times that the lipidic system is processed within the homogenizer setup. A key role is also played by the initial properties (and factors) associated with the processed sample, including the lipids’ (and bulk medium’s) composition and ionic strength, and the initial liposomes’ size-distribution and lamellarity. The major drawbacks of method are connected with the use of very high (operating) pressures and possible metal and oil contamination.

## 5. Novel Technologies for Liposome Preparation

The main drawbacks of conventional liposome formation approaches include the difficulty in achieving an easily scalable process for (large-mass) production, and the difficulties of obtaining elevated encapsulation efficiencies. Moreover, conventional methods may be not suitable for the processing of many (bio-) molecules, as they undergo structural (or functional) perturbations/alterations, due their exposure to detergents, organic solvent residues (with sensitive toxicity), and (high) shear homogenization (or sonication) processes, which may severely affect the clinical applications. With the aim of overcoming those critical issues, recently, novel technologies were developed for the production of liposome nano-formulations.

### 5.1. Freeze-Drying (Lyophilization) Method

Water-soluble drugs with lipid-based nanoformulations are generally subjected to leakage during preparation and storage on the shelf. Moreover, active drugs may be degraded because of possible oxidation phenomena and other chemical reactions before their use in drug-delivery applications. Those circumstances represent limiting factors in the commercial development of liposome nanoformulations. An approach to overcome these problems consists in the removal of water (ice) from the liposome systems in the frozen state (and at low pressures) [65,100].

**Figure 7:**
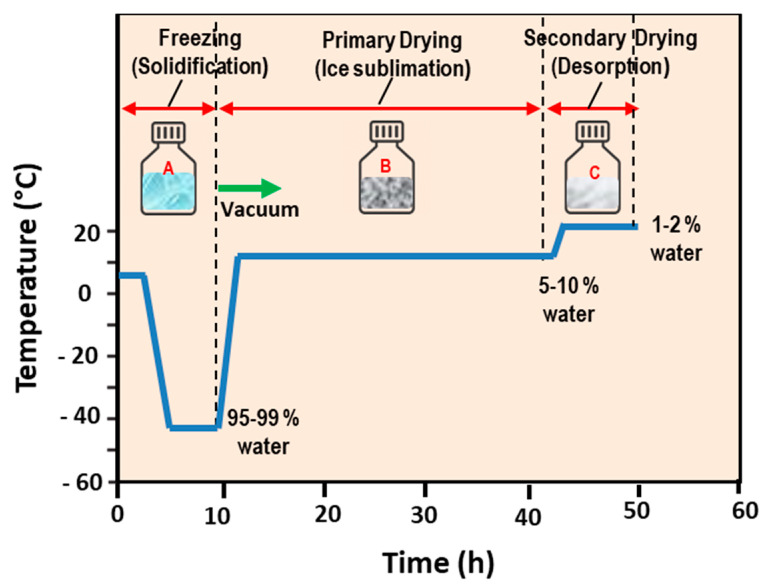
Schematic representation of the main stages of the freeze-drying (lyophilization) method. After the loading of the sample container (in flasks/vials), the system undergoes an initial freezing at atmospheric pressure, which is characterized by the formation of ice crystals (**A**), followed by a primary drying (ice crystal sublimation) (**B**) under vacuum. A secondary drying under vacuum favors the desorption of unfrozen water (**C**). Finally, the sample (product in vials) undergoes a backfill and stoppering process under partial vacuum, followed by the removal of the dried product from the freeze dryer.

**Figure 8:**
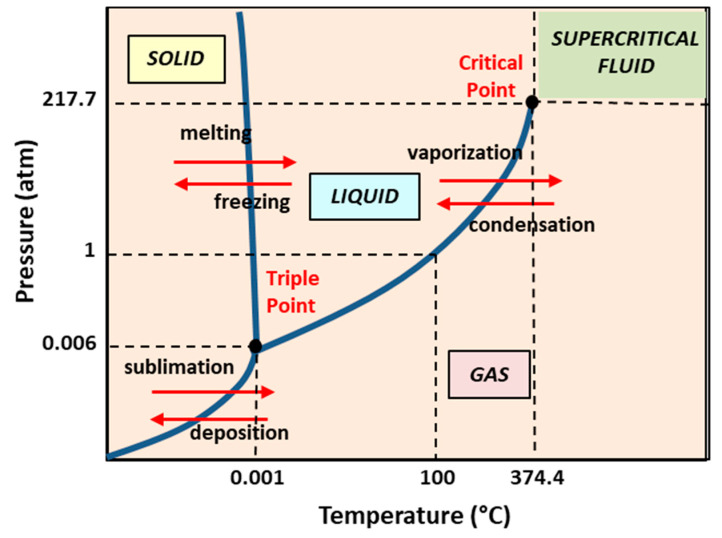
Phase diagram of carbon dioxide.

The freeze-drying method consists in the freezing of the aqueous solution containing the liposome formulation and the successive removal of ice by sublimation. In Figure 7, a schematic representation of the main stages of the freeze-drying (lyophilization) method is reported. Sublimation, i.e., the phase change when solid (ice) directly passes to a vapor phase without first passing through a liquid (water) phase, requires heat energy (and low pressures) for the frozen product to take place (Figure 8). In the freeze-drying method, the product is initially frozen (usually in a vial or a flask) at atmospheric pressure, and then placed under (a deep) vacuum, well below the water triple point. Finally, heating is applied in order to cause the ice to sublime (dry process). After the primary drying (sublimation), a secondary drying (under vacuum) is necessary (for the desorption of unfrozen water), followed by the removal of the dried product from the freeze dryer.

As lyophilized liposome products are extremely hydroscopic, in order to prevent rehydration from atmospheric exposure, they are sealed in airtight containers following freeze drying. For this reason, freeze dryers can be engineered with a “stoppering” capability by using vials with partially inserted stoppers. This approach allows for sealing the liposome products while they are still under partial vacuum (inside the processing unit). A backfilling with the dry nitrogen (inert gas) before the products’ sealing (or stoppering) is, finally, performed [100,101]. Although water is the main solvent that must be eliminated from the liposomes solution by the freeze-drying process, there are several nanoformulations that are manufactured via freeze-drying processes that require the use of organic co-solvent systems.

Frozen products present a crystalline or an amorphous glass nanostructure. While crystalline materials present a “eutectic” freezing/melting point (called “collapse temperature”), the amorphous formulations exhibit a corresponding “glass transition” temperature and, for this reason, are difficult to be processed with the freeze-drying method.

The freeze-drying method is very useful for the preservation of the shelf stability of the liposome systems, since water can favor unwanted chemical reactions, thus leading to the modification (or even degradation) of the drugs contained in the nanoformulation. The approach is suited to dry thermo-labile liposome products that would be degraded by the heat-drying process. This method, therefore, preserves a large variety of heat-sensitive biomaterials, including proteins, pharmaceuticals, tissues, and plasma components. The lyophilized form of the lipid-based pharmaceuticals ensure an increased shelf-life, especially when the inclusion cargoes are given by drugs that are not stable in the aqueous phase [100,101].

With the aim of ensuring the (cryo-) protection of the liposomes’ structure, during the freezing stage of the lyophilization process, sugar macromolecules such as sucrose, lactose, and trehalose are usually incorporated to the liposome systems [100,101,102,103,104,105]. In this case, during the use of the lipid nanoformulation, upon a rehydration process, the water molecules are able to replace the sugars and liposomes reconstituted without significant changes to their size. Certain sugars (such as trehalose) are able to mimic the presence of water, and are capable of preserving the integrity of dry liposomes and membranes [104]. Li and Deng [100] performed a freeze-drying process on a phospholipid t-butyl alcohol water–sucrose solution by initially freezing at the temperature T = −40 °C (for 8 h), successively drying (for 48 h at the same temperature), and finally drying the product for 10 h at T = 25 °C. They evidenced that the liposomes’ size and polydispersity were decreased with the increased sucrose concentration, while the dry liposomes could be stored in a sealed container for a long period [100]. Those results confirm, in part, the previous observations of Kiselev et al. [102,103], who observed a decrease of the vesicles polydispersity, and increases of the vesicles lifetime in an aqueous solution, extruded DMPC vesicles upon the addition of sucrose (in the concentration range of 0–40% *w*/*w*). In that study, no effects were detected on the membranes’ thickness and the hydrocarbon chains’ packing. It is worth noticing that the degree of water absorption strongly depends on the hydrophilic character of the phospholipid’s head group and, in part, on the specific composition and length of the hydrocarbon chain [102,103]. Liposomes formed with this method have a small size (<200 nm); with the use of suitable cryoprotectants, a highly efficient encapsulation (80%) with high stability and reproducibility can be obtained [102,103].

### 5.2. Dense Gas Technology: Supercritical Fluid-Assisted Methods

Traditional methods of liposome preparation have various shortcomings connected with the poor stability, high polydispersity, and high amounts of residual organic solvent. In particular, organic solvents used in the conventional preparation of liposomes may degrade the encapsulated active ingredients and may represent a cause of toxicity for human health and the environment. In this respect, the peculiar properties of dense gases have been used to substitute many organic solvents, thus enabling novel processing approaches, including purification (separation) and size-reduction processes. Dense gases in the region above the critical point, in fact, have mass transport characteristics similar to gases, and dissolution characteristics similar to liquids and traditional solvents [106,107]. More specifically, supercritical fluids are (non-condensable) very dense fluids at temperatures and pressures above the critical values (i.e., at the point where we observe the disappearance of the line between the gas and the liquid phase).

Several new liposome formation methods employ a supercritical fluid (SCF), i.e., a fluid maintained under supercritical temperature T_c_ and pressure P_c_ (see Figure 8). In this state, SCFs are excellent solvents for many lipids components. The variations in their temperature (or pressure) lead to sensitive modifications in their density and facilitate the solubility of a large variety of active components in the SCF. For those main reasons, SCFs are progressively replacing the organic solvents, as they favor a high-performing purification and separation procedure. [106,107]. More specifically, supercritical carbon dioxide (SC-CO_2_) represents a widely used organic solvent substitute, and is one of the most widely used non-toxic and environmentally friendly dense gases. Moreover, it has values of the critical parameters (i.e., temperature T_c_ = 31.1 °C and pressure P_c_ = 73.8 bar) that are easily accessible for a large variety of labile bio-materials.

The general supercritical fluid method is characterized by two stages [106,107]. Initially, in the high-pressure (P = 250 bar) part, the dissolution of the lipids in supercritical carbon dioxide is performed. Successively, the obtained (supercritical) homogeneous solution is successively expanded at the temperature of T = 60 °C, with the addition of (a small amount, of about 7% *v*/*v*) of ethanol. Interestingly, the ethanol total amount is 15-times lower than in the ethanol injection method. The expanded liquid is then mixed (injected through a nozzle) with a water phase, and liposomes (with encapsulated water-soluble drugs) are formed. This method produces large liposomes with sizes in the range of 0.2–4 μm, while only 3% of the liposomes are usually subjected to degradation [107,108]. In a variation of this method, the phospholipid(s) are initially mixed, while the SCF and the co-solvent mixture with the aqueous phase is successively decompressed by spraying it (through a nozzle) [108].

#### 5.2.1. Supercritical Reverse-Phase Evaporation (SC-RPE) Method

Otake et al. [109] reported the first supercritical reverse-phase evaporation technique, using SC-CO_2_ as the solvent for the phospholipids. The employed experimental setup is schematically reported in Figure 7 [109].

**Figure 9:**
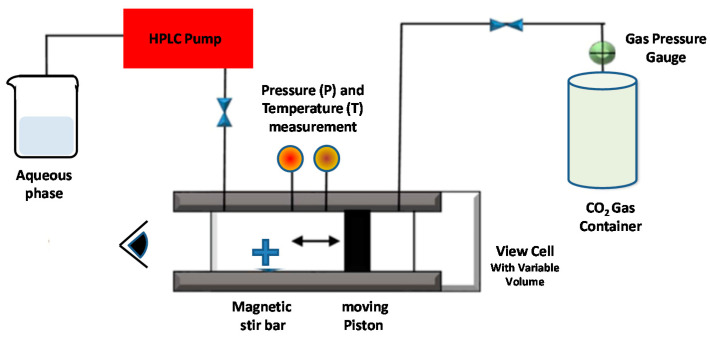
Schematic representation of the apparatus used in the supercritical reverse-phase evaporation method [109].

A variable volume chamber was used to progressively decrease the pressure of the system. Initially, the lipids, the compressed gas, and an organic co-solvent were combined and inserted in a (variable-volume) viewing cell, and stirred with a magnetic tip above the lipid phase-transition temperature (Figure 9). The temperature was then raised above the specific (phospho-) lipid phase-transition temperature and the supercritical temperature of carbon dioxide. After a given time of equilibration, an aqueous solution (containing the drug’s molecules) was introduced to the viewing cell through a high-performance liquid chromatography (HPLC) pump. Finally, after the reduction of the pressure through the release of the compressed CO_2_ gas, a homogeneous dispersion of LUVs (0.1–1.2 μm) was formed [109].

The SC-RPE method is similar to the decompression method of Castor and Chu [108], since the lipid, aqueous phase, dense gas, and modifier are first combined and then depressurized (decompressed) to form liposomes. The main differences is that, in the decompression method, the depressurization is obtained by spraying the solution through a nozzle, whereas in the SC-RPE method, it is given by the release of the dense gas from a variable-volume cell containing the sample.

An investigation of Imura et al. evidenced that, by varying the pressure and organic co-solvent concentration, it is possible to control the size and trapping efficiency of the formed liposomes [110]. More specifically, liposomes prepared at pressures below 200 bar are resultingly larger than those prepared at >200 bar. The minimum ethanol concentration to obtain water in a CO_2_ emulsion at 200 bar is 6.8 wt %. This represents the “optimal” and the ethanol “limit concentration” to obtain large unilamellar vesicles with the highest trapping efficiency, while at ethanol concentrations less than 6.8 wt %, multilamellar vesicles are formed since the (osmotic) shrinkage velocities and trapping efficiencies are low [110].

Recently, liposomes of various phospholipids prepared using an improved SC-RPE method (with supercritical CO_2_) exhibited the formation of a unilamellar vesicle structure with loosely packed phospholipids, with high stability (for one month) at room temperature [111]. Moreover, the SC-RPE method ensured a maximum trapping efficiency of glucose of 36% (for 20 mM l-α-dioleoylphosphatidylcholine (DOPC)), compared to less than 10% using the traditional (Bangham) method [111]. The SC-RPE method (with supercritical CO_2_) was also used for the construction of a novel liposome system consisting of DPPC/Cholesterol/C16-Arg2 lipopeptides (LPs)/DSPE-PEG2000 (60/30/5/5), for the delivery of bovine serum albumin (BSA) protein [112]. The constructed liposome nanoformulation (with diameter of about 1000 nm) evidenced an enhanced colloidal stability (of 90% over 40 h) and up to 70% entrapment efficiency for BSA, which was six-times higher than that obtained with the Bangham method. Small-angle X-ray scattering (SAXS) and differential scanning calorimetry (DSC) experiments evidenced that some amounts of LPs induced structural changes and phase transitions in the DPPC lamellar structure, with an improvement of the nanocarrier properties [112]. Those studies evidence the possibility of developing novel organic-solvent-free liposome nanoformulations with desired performance, which allows for addressing important environmental concerns.

#### 5.2.2. Supercritical Anti-Solvent (SAS) Method

**Figure 10:**
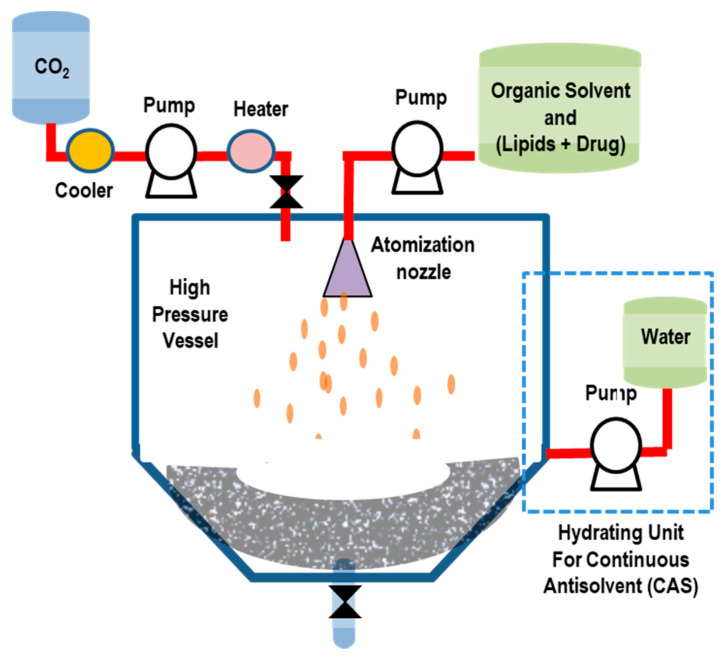
Schematic representation of the SAS method for liposome preparation. The SCF CO_2_ is pumped to the top of the high-pressure vessel until the system reaches a constant temperature and pressure. Subsequently, an organic solution containing the lipids and active substance is sprayed (through an atomization nozzle) as fine droplets into the above SCF bulk phase. Liposomes are finally formed in a successive hydration step. In the continuous antisolvent (CAS) method, the addition of the hydration unit allows for the hydration of the lipid suspension in the same autoclave under pressure.

In this method, a solution containing an organic solvent and the solute (lipids and active drugs) is placed in contact with a SCF, (such as SC-CO_2_) which is (completely) miscible with the organic solvent but acts as anti-solvent for the solute [113]. The dissolution of SC-CO_2_ in the liquid phase and the successive organic solvent extraction favors the precipitation of the lipidic nanoparticles. The processed solution is successively hydrated in an aqueous buffer solution, leading to the formation of liposomes. To remove any organic solvent in excess, a washing stage (with pure CO_2_) is finally performed. In Figure 10, we report a schematic representation of the SAS method for liposome preparation.

The SAS method represents, then, a relatively simple, efficient, and environmentally friendly process that enables very-low residual solvent contents. The SAS approach also allows for the processing of molecules with poor solubility, for the production of high quantities of liposomes [113]. With the SAS method, both hydrophobic and hydrophilic drugs (that do not need to be dissolved in SC-CO_2_) can be entrapped in lipid vesicles.

Lesoin et al. [113] used the SAS method to produce liposomes from lecithin liposomes and compared it with the TFH (Bangham) method. The liposomes produced with the SAS method exhibited a narrower and more reproducible particle size distribution (ranging from 0.1 μm to 100 μm). The encapsulation efficiency was, however, lower (10–20%) than that obtained using the TFH method (20%); the stability in suspension was low (after one month of storage at 4 °C) in both preparation methods. The SAS process was upgraded to the continuous anti-solvent (CAS) process, whereby the micronization and the successive liposome hydration process are performed in the same autoclave under pressure [114].

#### 5.2.3. Rapid Expansion of a Supercritical Solution (RESS) Method

**Figure 11:**
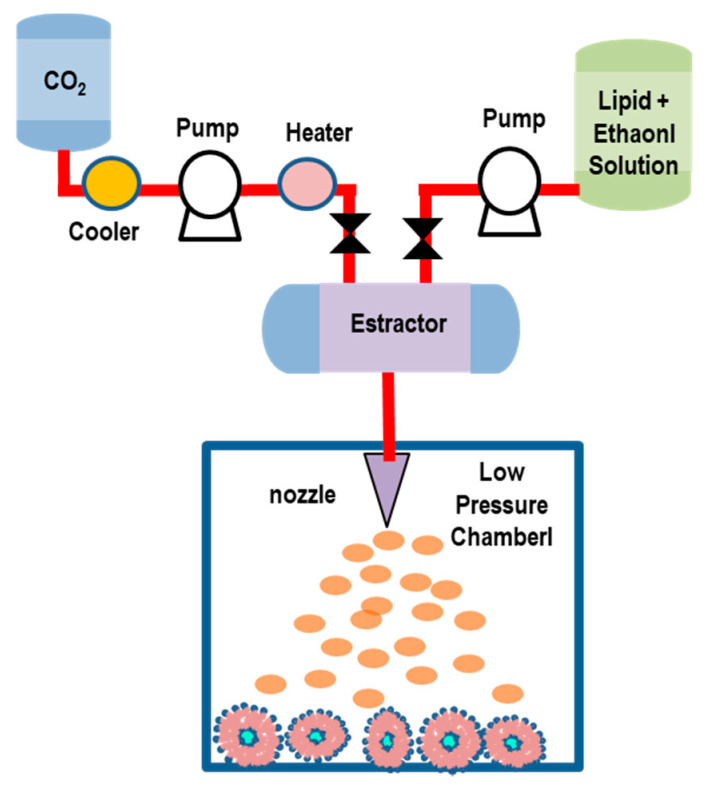
Schematic representation of the RESS method for liposome preparation. Initially, lipids are dissolved in supercritical CO_2_ and ethanol (5–10% of *v*/*v*) within an extractor. The resulting solution is depressurized through a heated nozzle in a low-pressure chamber. Finally, the formation of particles is generated, due to the supersaturation.

The RESS technique is carried out in two steps: initially, a solution containing lipids is dissolved in ethanol (5–10% of *v*/*v*) and supercritical CO_2_ within an extractor. The resulting solution is depressurized through a heated nozzle in a low-pressure chamber [115,116,117]. The rapid expansion/decompression (at supersonic speeds) through a nozzle favors the decrease of the pressure and the evaporation of CO_2_, thus leading to the supersaturation, and then, to the precipitation of the solid (that will be collected from the gaseous stream). In Figure 11, we report a schematic representation of the RESS method (adapted) for liposome preparation.

The rapid depressurization causes the lipids’ desolvation, which favors the formation of layers around the droplets, and finally, the formation of liposomes. The obtained liposomes exhibit a oligolamellar vesicle structure with a broad size distribution (ranging from 50 nm to nearly 1 µm). Either hydrophobic or hydrophilic drugs can be encapsulated by this method. The size (size distribution) and encapsulation efficiency (EE) of the liposomes can be regulated by modifying the pre-expansion pressure, the flow rate, and the nozzle position in the low-pressure chamber (reactor). The main drawbacks of this approach are connected with the poor solubility of most of the biomaterials (e.g., polymer-based macromolecules) in SC-CO_2_, and the difficulty of the separation between co-solvents and vesicles during the depressurization process, which causes a sensitive increase of the liposome production costs [115,116,117].

A modified RESS technique was applied to encapsulate an essential oil (extracted from Atractylodes macrocephala Koidz) into liposomal nanocarriers [118]. Lipid components and the drugs (essential oil) were dissolved in the mixture with SC-CO_2_/ethanol, and successively, the solution was sprayed into an aqueous medium through a nozzle to form a liposome suspension. By changing the expansion processing conditions (such as the temperature and pressure of SC-CO_2_ and the amount of ethanol), it was possible to control the performances of the formation process. Under optimum conditions (P = 30 MPa, T = 338 °K and 15% ethanol mole fraction in SC-CO_2_), the formed liposomes appeared as double-layered colloidal spheres (size = 173 nm) with a narrow size distribution, and an entrapment efficiency (EE) of 82.18% (and drug loading of 5.18%) [118].

The RESS method was recently used to produce melatonin-loaded liposomes (MLL) [119]. The results evidenced that 140 bar was the best pressure to obtain a maximum value of EE (82.2%). MLL characterizations, performed using infrared spectroscopy, transmission electron microscopy, light scattering, and gas chromatograph-mass spectrometry, evidenced an average nanocarrier diameter of 66 nm with a uniform size distribution. Stability tests evidenced that MLL maintained a good preservation duration, and residual solvent experiments indicated that only 1.03% (mass ratio) of ethanol remained in the products. Simulated release (through an in vitro simulated digestion experiment) indicated a slow release feature in early digestive stages and a more thorough characteristic in the late stages of digestion [119]. Finally, phosphatidylcholine (PC) liposomes encapsulating vitamin C (VC) were investigated by the rapid expansion of the supercritical solution process (RESS), through the combined effects of temperature, pressure, and feeding ratio of VC against the drug-loading content of the prepared liposomes [120]. Under optimal operating conditions (pressure of 25 MPa, temperature of 48 °C, and feed ratio of VC against PC of 0.25), the VC-loaded liposomes exhibited the loading content of 75.38 ± 1.03%, and well-defined and reproducible particle sizes (270.4 ± 5.2 nm), PDI (0.254 ± 0.010), and zeta-potential (−41.7 ± 0.9 mv) [120].

#### 5.2.4. Supercritical-Assisted Liposome Formation (SuperLip) Method

**Figure 12:**
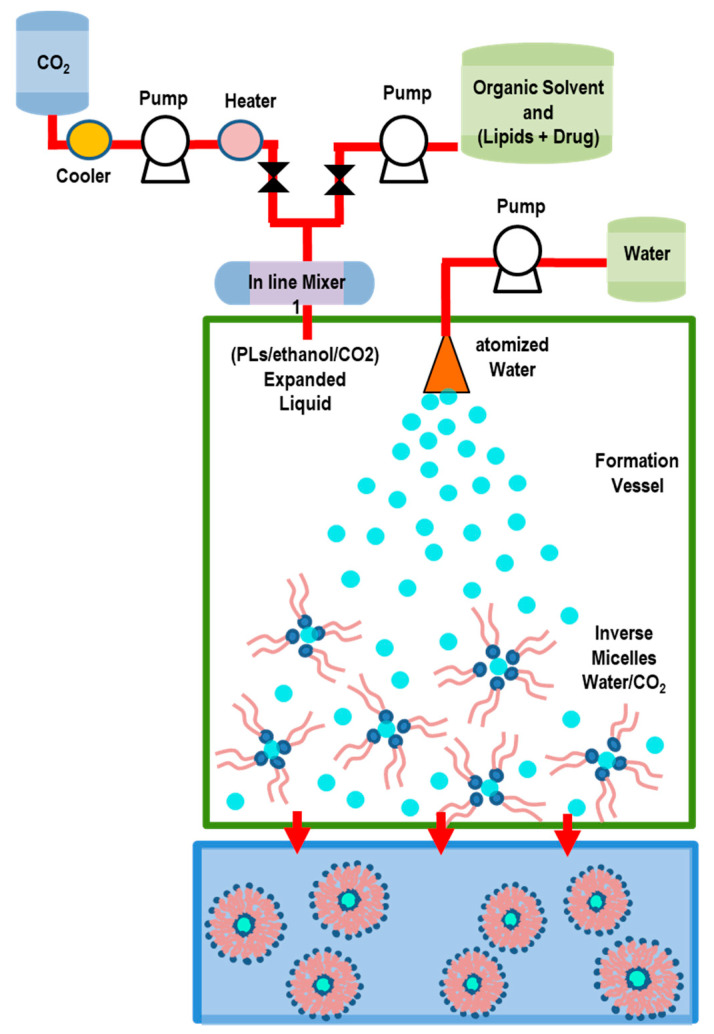
SuperLip method and mechanism of liposome formation. A high-pressure vessel is filled with an expanded liquid mixture (formed by PLs/ethanol/CO_2_ containing drugs). Water droplets are produced by atomization inside a high-pressure vessel. These droplets are rapidly surrounded by a lipid layer, forming a w/CO_2_ emulsion (inverted micelle). Finally, liposomes (*w*/*w* emulsion) are formed when they fall in the water pool located at the bottom of the vessel.

In the SuperLip method, the ethanol solution and CO_2_ are continuously fed to a homogenizer, forming an expanded liquid which is then delivered to a precipitation vessel. In this vessel, water with droplets containing drugs are produced by means of an atomization process (inside a high-pressure vessel). The droplets are then surrounded by a lipid layer (thus favoring the formation of w/CO_2_ emulsion) which falls into the water pool placed at the bottom of the vessel, where liposomes are formed (Figure 12) [121].

The SuperLip technique allows for a sensitive control of the liposomes’ size (in the nanometric and sub-micrometric range) and size distributions with a sensitive reduction of the residual solvent. Moreover, the high encapsulation efficiencies (from about 80% up to 99% for hydrophilic compounds) allow for the entrapment of a large variety of active drugs, including antibodies, proteins, antibiotics, antioxidants, essential oils, and dyes [121]. The entrapment of additives (such as cholesterol and phosphatidylethanolamine) on the lipidic double layer compartment of liposomes allows for obtaining a more compact structure with encapsulation efficiencies of 96% (cholesterol) and 95% (phosphatidylethanolamine) [121]. The versatility of this method may stimulate the industrial applications in a large variety of technology fields, such as pharmaceutics, cosmetics, textiles, and nutraceuticals.

By using the SuperLip process, lutein (a lipophilic ophthalmic nano-drug) has been encapsulated in liposomes with mean diameters between 153 ± 38 and 267 ± 56 nm, with high lutein encapsulation efficiencies (between 86.5 ± 0.4% and 97.8 ± 1.2%) [122]. The variation of temperature for the production of liposomes showed a significant impact on lutein retention time (within the double lipid layer). Lutein drug release from liposomes produced at 35 °C ended after almost 4.5 days, whereas liposomes produced at 40 °C showed a faster lutein release in 3 days. Then, vesicles obtained at 45 °C released their lutein content in only 2 days [122]. Moreover, the SuperLip method has been employed to produce ampicillin-loaded liposomes that were successively entrapped into alginate gels, while a final treatment with supercritical CO_2_ drying allowed for obtaining an aerogel [123]. This combined approach allowed for obtaining a meta-carrier (i.e., a carrier within another carrier) with a drug release that works with two mass-transfer resistances (in series). The first mass resistance was given by the structure of the aerogel, during the release of ampicillin not entrapped into liposomes. The second mass resistance was given by the lipidic double layer of liposomes. The initial liposomes (with a diameter of 200 ± 77 nm and EE of 69.5 ± 1.2%) were successfully entrapped into the aerogels (as confirmed by EDX analysis), while the antibiotic entrapped into simple liposomes alone was totally released after about 3 days. The antibiotic entrapped into a meta-carrier reached a plateau after about 5 days [123].

#### 5.2.5. Depressurization of an Expanded Liquid Organic Solution into Aqueous Suspension (DELOS) Method

**Figure 13:**
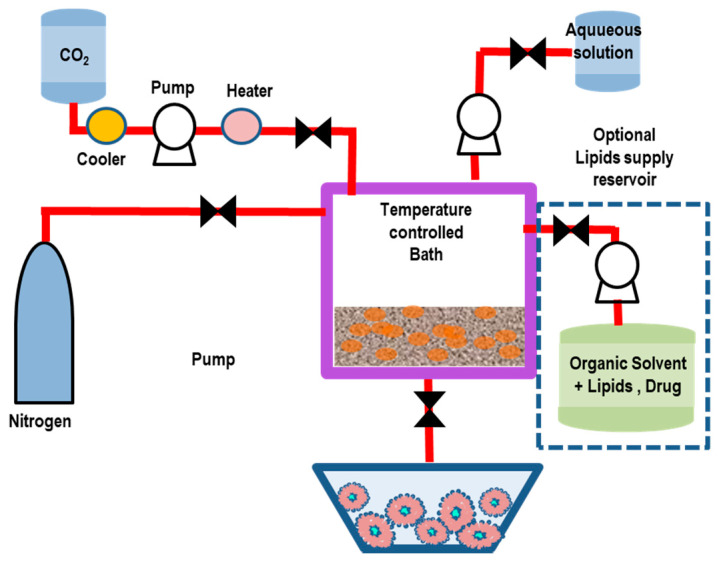
Schematic representation of the DELOS method. The lipids dissolved in organic solvent contained in a vessel at desired temperature and pressure, are mixed with SC-CO_2_ (used as a co-solvent to the organic solvent). Then, the mixture (depressurized at 35–55 bar) is expanded into CO_2_, and injected through a nozzle into in a vessel containing water bath and active drugs, where the liposomes are formed.

In the DELOS method [124,125], the lipids (and active drugs) are first dissolved in an organic solvent (such as ethanol) contained in a vessel at fixed temperature and pressure, and then mixed with SC-CO_2_ (used as a co-solvent). The mixture (depressurized at 35–55 bar) is then expanded into CO_2_, and (through a nozzle) is successively injected in a vessel containing water bath and active drugs. The evaporation of part of ethanol allows the contact between lipids (transported by bubbles) and water, and favors the formation of liposomes. (Figure 13). The main advantages of the DELOS method are that the resulting liposomes are small in size, have relatively high uniformity and homogeneity (of spherical shape), and enhanced colloidal stability, as well as the possibility of reducing the usage of sterols [124,125]. However, the residual ethanol concentration does not always ensure the full biocompatibility and safety of the liposome nanoformulation products [124,125].

The engineering of new (pH-sensitive) lipid nano-vesicles, for the delivery of miRNAs (and other small RNAs) for the triggering of a suppressive response into tumor cells, was recently reported [126]. The nanocarriers, developed by means of the DELOS technique, exhibited an enhanced stability upon storage that preserved their (unilamellar) morphology, size (<150 nm), and low polydispersity (<0.25), for at least up to 6 months. The functionality of the developed pH-sensitive nanocarrier was tested for miRNA delivery against selected neuroblastoma (a common aggressive extracranial solid tumor in children). The high stability, together with their simple and effective (one-step, green, and scalable) manufacturing procedure, makes these sRNA nanocarriers highly attractive for the translation to the clinical phases, compared to other nanocarriers with low stability and with challenging (multistep) scalability [126]. Recently, the DELOS method has been employed to prepare α-galactosidase-loaded nanoliposomes (nanoGLA) for the treatment of Fabry disease (a lysosomal storage disease that arises from a deficiency of the enzyme α-galactosidase A (GLA)) [127]. Within the investigation, a nanoformulation for (preclinical) in vivo studies was developed by implementing a Quality by Design (QbD) approach. This methodology allows for developing efficient drug-manufacturing and control methods. Through a risk analysis and a Design of Experiments (DoE), it was possible to obtain the critical parameters (such as the GLA and lipid concentration) for achieving a stable nanoformulation, and to optimize the production process for in vivo preclinical testing [127].

In conclusion, apart from being organic solvent-free methods, the supercritical fluidic methods offer, in general, many other advantages—such as the use of CO_2_ as a cheap and environmentally harmless solvent, the possibility of controlling particle size, in situ sterilization, and large-scale production. However, the disadvantages of the supercritical fluid technique are mainly connected with the high cost, low yield, and the use of high pressures (i.e., 200–350 bar), which require special infrastructures. Those circumstances discourage their applications for the industrial development of liposomal technologies.

### 5.3. Microfluidic (Channel) Methods

In the microfluidic method, lipids dissolved in ethanol (or isopropanol) solvent, are successively propelled within microscopic channels (with 5–500-μm cross-section) [128,129,130]. The alcoholic phospholipids solution, focused between two aqueous streams in a microfluidic channel (microchannel), generate a (hydrodynamic) laminar flow and a (diffusive) mixing at the two (liquid) interfaces that favors the lipids’ self-assembly into vesicles. With the precise control of mixing and the fluid flow rates, this method allows for the production of small (monodisperse) liposome nanoformulations with controllable sizes and distributions, with the use of low-toxicity solvents (such as ethanol). Compared to traditional bulk methods, the final product does not require post-production processing (i.e., extrusion, sonication). Although the microfluidic method offers high versatility and flexibility, the main disadvantages of this approach are connected with use of organic solvents, sensitive mechanic agitation, and the difficulty of large-scale production [128,129,130].

In a recent investigation of Jahn et al. [129], the liposome system (consisting of the lipids DMPC, cholesterol, and dihexadecyl phosphate (DCP)) formed by the microfluidic process evidenced that, by modifying the alcohol-to-aqueous (volumetric) flow rate ratio, the vesicle sizes (and distributions) were tunable over a mean diameter (between 50–150 nm). The authors also observed that the formation of liposomes strongly depends on the (focused) alcohol stream width and its mixing with the aqueous stream [129].

**Figure 14:**
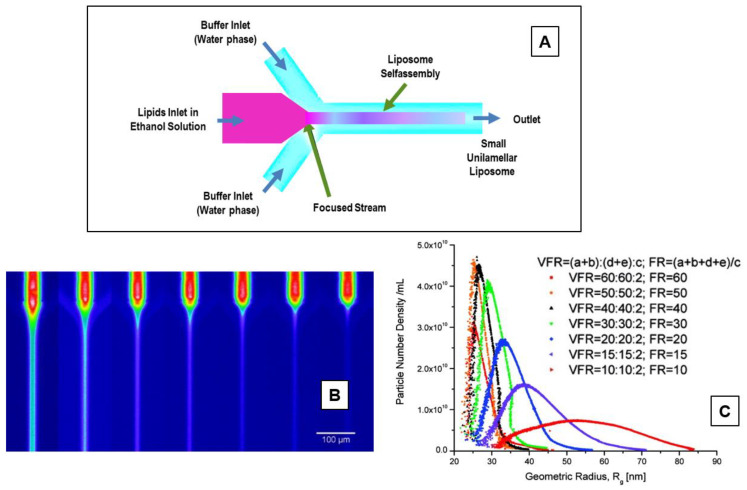
Schematic representation of a three-inlet microfluidic setup, (**A**). Confocal microscopy (false-color) pictures of the hydrodynamic focusing of an isopropyl alcohol (IPA) stream (that contains sulforhodamine (**B**) by two (aqueous buffer) streams, for 7 flow rate ratios (FRRs), increasing from 5 to 35 (increments of 5 from left to right) at a total constant volumetric flow rate VFR = 100 μL/min (**B**). Final liposome size distribution at different FRRs (**C**). Adapted with permission from Jahn et al. [129], Copyright 2007 American Chemical Society.

In Figure 14A, we report a three-inlet microfluidic setup representation, illustrating the (SUV) liposome self-assembly. We also report (Figure 14B) the confocal microscopy images evidencing the hydrodynamic focusing of an isopropyl alcohol (IPA) stream containing sulforhodamine B, by two adjacent aqueous buffer streams (not visible), for 7 different flow rate ratios (FRRs). Finally, the corresponding liposome size distributions at different FRRs are reported in Figure 14C [129].

The effects of the nano-formulation parameters on the colloidal stability and pharmaceutical properties of nano-liposomes remotely loaded with dexamethasone were recently investigated by using the microfluidic and the TFH methods [131]. The liposomes generated by the microfluidic method showed a unilamellar structure, while the liposomes produced by the TFH technique were multilamellar. Under the same remote loading conditions (using a calcium acetate gradient), both formulations released the drug for almost one month, while a higher loading capacity and low batch-to-batch differences were observed for liposomes obtained by the microfluidic method. In vitro studies showed that both formulations exhibited a non-toxic behavior, associated to human adult retinal pigment epithelial cell line-19 (ARPE-19) cells, and efficiently reduced the inflammation, with the liposomes obtained by the microfluidic technique slightly outperforming [131]. Those results evidence that the microfluidic technique offers advantages for the generation of liposomal nanoformulations with an enhanced drug-controlled release and biopharmaceutical profile and with high scalability.

In the last decades, a variety of new microfluidic methods have been developed for the formation of liposome nanoformulations for biomedical applications. Among many variations of this method, the micro hydrodynamic focusing (MHF) method developed by Jahn et al. [129] is able to produces(40–140 nm) homogeneous SUVs and LUVs with excellent control of the flow and mixing conditions. Moreover, high-throughput novel microfluidic architectures can be engineered for the mass production of liposome nanoformulation. Finally, on-chip liposome loading with (both hydrophilic and lipophilic) bio-active compounds represent an exciting research field to develop using this method [129,130].

In the microfluidic droplets (MD) method, two immiscible phases (such as water and oil) are forced to flow (under specific conditions) into a microchannel and generate (uniformly sized) small droplets of one phase. With this method, the dissolution of lipids in hexane allow for the formation of giant (4–20-μm) liposomes. In the work of Shum et al. [132] a water-in-oil-in-water (w/o/w) double emulsion was prepared by the microfluidic technique with the lipid dissolved in the central oil phase (a volatile mixture of toluene and chloroform). By evaporating the organic layer, the monolayers transform, in lipid bilayers, into giant vesicles [132].

**Figure 15:**
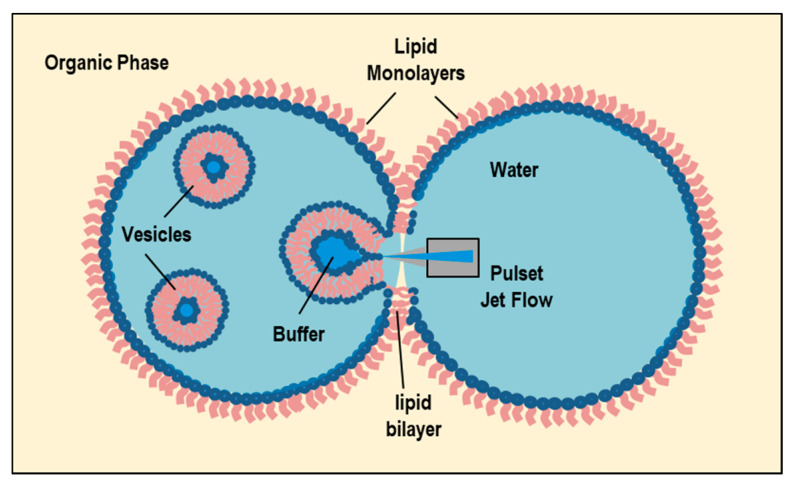
Schematic representation of the pulsed jet flow method. The lipids initially dissolved in the organic phase self-assemble (at the oil–water interface) as monolayers. The periodic pulses of a fluid jet (of a buffer) is directed at the interface of two nearby W/O microemulsions, and favors the formation of giant unilamellar vesicles [133].

The pulsed jet flow microfluidic method [133] (Figure 9) consists in drying the solution containing phospholipid in microtubes (micro-capillary). The formed lipid film is then hydrated (within the microtubes) through a perfusion process that forms giant (200–500-μm) vesicles of uniform size with high encapsulation efficiency. The lipid bilayer is formed by bringing in contact two aqueous drops coated with a lipid monolayer within an oil phase containing the phospholipids. The draining of oil film between the two drops favors the formation of a planar bilayer, into which a periodic pulse of a fluid jet of aqueous solution (buffer) is injected (using a micro-dispenser). The pulsed jet flow, directed in the interface region between the two water drops, results in the formation of giant vesicles (Figure 15). One limitation of this method is connected with the difficulty of automation process, as the control of the position of the microcapillary (close to the bilayer) should be performed manually [133].

Recent innovations of the microfluidic technique includes the continuous flow liposomes formation, based on the transmembrane pH (or ion) concentration gradient, which is created by using an on-chip microdialysis membrane [134,135]. By using a thermoplastic microfabrication method, it has been possible to develop fully integrated microfluidic devices that favors a low-cost (scale-up) technology for the production of liposomal nanocarriers, in a (continuous) flow process. This integrated method (called pharmacy-on-a-chip) allows for the large-scale production (at about 100 mg/h lipid) of a new generation of fully optimized, multi-agent, and targeted liposomal nanoformulations [134,135].

### 5.4. Membrane Contactor Method

**Figure 16:**
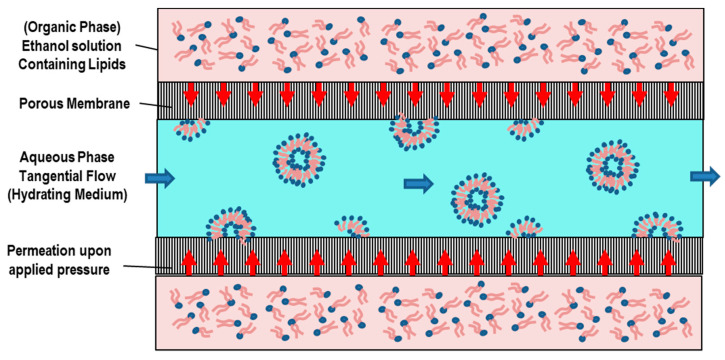
Liposome formation process in the membrane contactor method.

The membrane contactor technique is based on a modified ethanol injection method. The reference setup consists of two pressurized vessels, one for an organic phase containing lipids, and the other for an aqueous phase, separated by a special porous glass membrane, having pore sizes that allow for the flow of the organic phase [68,136,137]. Often, the (alternative) polypropylene hollow fibers are employed as the membrane, as they allow for larger areas and uniform flows. A lipid phase dissolved in alcohol (such as ethanol), is pressed through the porous membrane and extruded into the aqueous phase, which flows in a tangential direction to the membrane surface (Figure 16). Upon the contact between the organic solution with the aqueous phase flow, the lipid molecules self-assemble into liposomes, and are finally collected at the exit portal of the membrane. At the end of the process, the ethanol is removed by rotary evaporation (under reduced pressure), while the generated liposomes can be stabilized by magnetic stirring. The porous membrane module can be regenerated by washing (flushing) with a water/ethanol mixture [65,136,137,138].

The advantages of this new technique are the high EE of drug molecules and the precise control over the mean size (and size distribution) of the prepared liposomes by tuning the process parameters (such as the lipid concentration, aqueous-flow rate, and pressure of the organic phase) [136,137,138]. Moreover, the scaling-up abilities of the method allow for continuous, large-scale (multilamellar) liposome preparation for industrial production purposes.

A novel method based on the use of a polypropylene hollow fiber module has been successfully used for the formation of liposomes filled with a hydrophobic drug model, namely, spironolactone (used for pediatric medication application) [137]. The initial liposome suspension was composed of phospholipids (DPPC, EPC-3 or Lipoid^®^ E80, and cholesterol (20%, *w*/*w*)) dissolved in 250 mL ethanol. Transmission electron microscopy experiments evidenced the formation of spherical oligo-lamellar vesicles with a mean size of 113 nm (and zeta potential of ζ = −43 mV) for drug-free, and 123 nm (ζ = −23 mV) for drug-loaded liposomes. The entrapment efficiency was high (93%), while the (rapid) release profile showed a complete release within about 5 h, and good stability for 2 months. The study also evidenced a liposome size (distribution) decrease with the decrease of the organic phase pressure and the phospholipid concentration, and the (relative) aqueous phase increases [137]. A novel application of micro-engineered membranes (using nickel micro-engineered flat disc membranes with a uniform pore size of 5–40 μm and a pore spacing of 80 or 200 μm) was used for the investigation of the process parameters during the encapsulation of vitamin E in a liposome system composed of POPC or Lipoid^®^E80 and cholesterol stabilizer [138]. Under optimal conditions, TEM images evidenced the spherical multi-lamellar structure of vesicles with a mean size of 84 nm (for Lipoid E80) and 59 nm (for POPC), which increased with increasing membrane pore size and decreasing pore spacing. A high entrapment efficiency of 99.87% was achieved when Lipoid E80 liposomes were loaded with vitamin E. Lipoid E80 (or stearic acid) liposomes stabilized by cholesterol maintained their initial size within 3 months, while the reproducibility of the formation technique was high. These results evidence that the hollow fiber module-based technique has potential for the (fast) continuous production of nanosized liposome suspensions at large scales [138]. In Table 1, we report the main features obtained by using different formation methods.

## 6. Drug Loading in Liposome Nanoformulations

The main aim of the liposome formation process is to create nanoformulations dedicated to the efficient transport and delivery of lipophilic (within the lipid bilayer), hydrophilic (in the aqueous core), and amphiphilic (partitioned at the bilayers surface) drugs. The amount of drug loading depends not only on the method used for liposome preparation, but also on the drugs’ chemical characteristics and on the composition and physicochemical properties of the liposomes (such as lipids’ concentrations and charge characteristics, drug/lipid ratios).

In the passive loading method, drugs are loaded during the liposomes’ preparation. This method can be performed by means of solvent dispersion, mechanical dispersion, and detergent removal methods [65,68]. For example, in the thin TFH method, the lipophilic drugs are incorporated (in large amounts) within the lipid bilayers during the first stages of the liposomes’ self-assembly process. On the contrary, the hydrophilic drugs, which are usually incorporated during the (aqueous buffer) hydration stage, present lower incorporation efficiencies, as only part of the drugs are present within the liposome core region, while part of them still remain in the hydration volume (outside the liposomes). For this reason, strategies can be developed to increase the aqueous phase within the core volume, by means of the different methods of liposome formation.

Active loading methods, which incorporate drugs after liposome preparation, generally exploit the diffusion properties of a gradient which is established across the surface of the liposomes (gradient loading) by involving the addition of suitable buffers. Active loading can be obtained with lyophilization, whereby the formed liposomes are generated by transfer of the lipids from organic to aqueous phases [65,80]. The use of active-loading approaches generally shows higher EE as compared to the passive methods. Particularly interesting is the (transmembrane) pH-gradients method, as it can be used to encapsulate a variety of active drugs (including anticancer, anti-anesthetics, antimalarials, etc.). Interestingly, several amine-functionalized drugs (including doxorubicin, daunorubicin, chlorpromazine, quinine, quinidine, dopamine, serotonin, and chloroquine) exhibit a pH-gradient effect after liposome preparations by accumulating protons in the vesicles’ interior (and exhibiting an acidic character in their internal region). In order to create a pH gradient across the liposome membrane, the pH of the exterior aqueous compartment can be increased through the incorporation of an alkylating agent (alkaline buffer) or by exchanging (by gel filtration or dialysis methods) the external media with the desired buffer. According to this approach, the liposomes are first hydrated with a given buffer (of a fixed pH), followed by dialysis in an excess of another buffer (with a different pH), in order to replace the buffer in the liposomes outside region (and to create a trans-membrane pH gradient). In this case, uncharged drugs entering the liposomes become charged inside the first buffer (of the liposome aqueous core) and remain entrapped in the liposomes (as the charged drugs cannot diffuse through the lipid layer) [65,68].

## 7. Post-Formation Processing of Liposomes

### 7.1. Purification of Liposomes Nanoformulations

Irrespective of the adopted formation method, non-encapsulated compounds (such as non-entrapped drugs, small molecules, or contaminant molecules) are generally present in the external (liquid) environment of the generated liposomes, and must be removed through a purification process from the final nanoformulation. The liposomes purification process represents, then, a further stage of the liposomes’ manufacturing process. The main techniques employed for the removal of the non-encapsulated materials include the ultra-filtration, ultra-centrifugation, dialysis, and (size exclusion, gel-permeation, or ion-exchange) chromatography. Due to column equilibration and dilution processes connected with those techniques, the purification process requires lots of time and can cause a sensitive decrease of the final liposomes’ production.

Another very important step of the post formation process is connected with the removal of residual organic solvents from the final liposomes. The use of the organic solvents (such as ethanol, methanol, chloroform, ether, and methylene chloride) represents a crucial stage in the liposomes’ formation processes, as it facilitates the molecular dispersion process of lipids and prevents the oxidation during the lipids’ storage. However, the residual solvents which are present in the final products may destabilize the liposomes, thus posing some risks upon application [65,68]. Although the (organic) solvents are usually removed by evaporation techniques, this process causes a concentration of the lipids (and of unwanted the contaminants) in the residual solvents that is not easy to further remove. For this reason, the manufacturer should clearly indicate (on the nanoformulation specifications) the residual levels of solvents in the final product (and the allowable safety level).

Finally, liposome nanoformulations should be protected from oxidation. Most of the lipid nanocarriers contain unsaturated lipids (acyl chains). During preparation, storage, or normal use, unsaturated lipids may undergo oxidative degradation (lipid peroxidation), a chemical process that involves some free radical reactions with the formation of cyclic-peroxides and hydro-peroxides. The lipid peroxidation process may be minimized by protecting them by keeping them under inert gases such as nitrogen or argon (in order to have minimal exposure to oxygen). Peroxidation can be minimized also by keeping liposome formulations in light-resistant containers or by the removal of heavy metals (adding ethylenedinitrilotetraacetic—EDTA). Finally, the addition of antioxidants such as alpha-tocopherol or butylated hydroxytoluene can also minimize lipid oxidation processes [65,68].

### 7.2. Sterilization of Liposomes

As parenteral route is the most frequent way of administration, liposomes nanoformulations should be free of viable microorganisms (such as bacteria, fungi, spores, etc.) that are capable of altering their properties. For this reason, it is important to remove all possible microorganisms through a sterilization process. The sterilization process can be achieved through various approaches, including steam heating (autoclaving), ultraviolet and gamma ionizing irradiation, chemicals, and filtration methods. However, as liposomes have high sensitivity and a tendency suffer from (physico-chemical) alterations, the efficient use of sterilization processes still remains a difficult challenge [139]. We will briefly analyze the main characteristics of the sterilization processes, highlighting their critical aspects.

The steam (autoclaving) sterilization method consists of the combination of saturated steam under heat and pressure that causes the germs’ destruction by hydrolysis of proteins. Many investigations have evidenced that this method is responsible for several liposome alterations that involve the oxidation and hydrolysis of lipids, phase transition and aggregation, and degradation (or leakage) of the encapsulated drugs. For this reason, this technique is suitable only for a limited number of liposome nanoformulations.

Heat sterilization may cause structural phase transitions (and correlated degradations and/or drug leakages), as well as the oxidation or hydrolysis of the component phospholipids (at higher temperatures (T > 121 °C)). For this reason, this method cannot be considered a reference method for liposome sterilization.

Although the gamma ionizing irradiation has a high-energy ionizing power (and then a strong penetration capacity), the sterilization process with this radiation may cause liposomes’ degradation by lipid peroxidation (of unsaturated lipids), hydrolysis or fragmentation of component lipids, and changes in pH. On the contrary, unlike gamma rays, ultraviolet (UV) radiation is a non-ionizing (low-energy) radiation with poor penetration capacity in materials, and for this reason, is not effective to cause the sensitive sterility of liposomes.

Ethylene oxide (chemical) sterilization uses ethylene oxide gas as a sterilizing antimicrobial agent whose sterilizing mechanism consists of the alkylation of the side chains of DNA, RNA, and enzymes, thus causing a strong metabolism inhibition and avoiding the multiplication of microorganisms. However, due to the flammable and explosive nature of ethylene oxide, and the toxic, carcinogenic, mutagenic character of its residues, the use of this sterilization method is very limited.

Sterilization by filtration is a relatively time-consuming method, based on the use of a sterile, disposable filtration unit consisting of an aseptic bacterial-free membrane (0.22-μm) or depth filters for the removal of the microorganisms present in gaseous or liquid products. This method, which is not applicable for liposomes greater than 0.2 µm, are ineffective for the filtration of smaller viruses or bacteria.

Aseptic manufacturing consists in the preparation and the filling of a product in a controlled sterile environment (class A environment) equipped with sterile materials and equipment. This method is quite complex and expensive, as several stages have to be performed in sterile environments. Additionally, the sterility level cannot be easily evaluated, and a potential risk of contamination (connected with initial raw materials that are not effectively sterilized) still remains.

In view of the drawbacks of all these conventional approaches for liposome sterilization, it is crucial to search for an alternative method in order to ensure the sterility of liposomes in a green, effective, and inexpensive fashion [139]. In this respect, an interesting alternative method for the liposomes’ sterilization can be connected with the use of the supercritical carbon dioxide (SC-CO_2_) technology [140]. This method allows for producing and sterilizing liposomes’ nanoformulations (in a single step). However, these approaches require further investigations and optimization using the standard biological indicators.

### 7.3. Microfluidic Lab-on-Chip Nanodevices for the Combined Formation, Drug Loading, and Purification of Liposomes

#### Micro- and Nanofabrication Techniques

The preparation of versatile nanoplatforms, based on microfluidic chips, have stimulated the development of multitask lab-on-chip devices for biomedical and nanomedicine applications [141]. With the successful development of the micro- and nanotechnologies’ fabrication processes, the liposome preparation techniques have been revolutionized with the development of novel smart devices. Recently, microfluidic (continuous-flow) formation of liposomes was combined with in-line drug loading and final sample purification [141,142,143].

Using on-chip microdialysis elements, it is possible to develop multitask systems that allow for the encapsulation efficiencies of a variety of lipids and that (through the creation of suitable transmembrane pH and ion gradients) favor the remote loading of drugs into the liposomes. The novel approach also allows for post-formation refinement, and for the determination of the liposomal nanoformulation properties (such as size, particle number, and surface potential). This novel microfluidic technology promises the precise control of the size and lamellarity of liposomes, and the reduction of the volumes (and costs) of the processed chemical components (and reagents), thus allowing for potential upgrades for the lab-on-chip-scale production and integration of process within analytical technologies [141,142,143]. In a recent investigation, a continuous-process flow system for the simultaneous preparation, drug loading, and purification of liposomes from contaminants has been developed [143]. The purification step, which is based on a tangential flow-filtration (TFF) device, allows for purifying a large variety of liposome nanoformulations (with very large recovery of lipids > 98%) and for the efficient removal of the non-entrapped compounds (>95%) and organic solvents (>95% reduction) in a very short time (i.e., less than 4 minutes). Those studies demonstrate the feasibility of the advanced on-chip formation and purification of liposome batches for development within industrial processes [143].

## 8. Characterization Methods of Liposome Nanocarriers

The behaviour of liposome nanoformulations in solution depends on their (nano-) structural organization at the molecular (and supramolecular) level. The performance of liposomes in biomedical and nanomedicine applications is strongly related to a number of control parameters, such as dimension, shape, morphology, lamellarity, and surface functionalization. In this respect, the structural characterization of the conformation of the underlying bilayer vesicles is crucial for the study of the performance of these nanocarriers systems, including their biodistribution and passive/active targeting capacity. For this reason, after their formation, liposome nanoformulations are subjected to characterization in order to evaluate the physico-chemical properties that influence their colloidal stability and their therapeutic and biological performances. More specifically, the main liposome properties should be investigated in specific solution conditions that resemble the standard conditions of administration. The study of the self-assembly processes involving lipid components (and amphiphilic molecules in general) represents also a crucial step and a relevant topic for the investigation of the interaction of liposomes (or nanoparticles) with biomembranes, and for the elucidation of various mechanisms connected with important physiological bio-processes involving biomembranes (such as phase changes, fusion, separation, biomembrane perforation, and endocytosis), as well as a variety of pathological processes regulated by specialized biomembrane nanodomains (lipid rafts) [144,145,146]. In the next section, we highlight the importance of some structural parameters of liposome systems, and shortly describe the main techniques used to measure them.

### 8.1. Small-Angle X-ray/Neutron Scattering (SAXS/SANS) and Diffraction Techniques

Among the experimental methods, the small-angle X-ray scattering (SAXS), small-angle neutron scattering (SANS), and diffraction methods are the most widely used for a non-destructive characterization of the structural properties of liposomes [147,148,149,150]. Scattering techniques also provide the fundamental tools for investigating the main interactions of nanocarriers in different solution environments, and for investigating conformational modifications and structural transitions which are relevant for the prediction of the structure–function relationship in many biological processes [151,152,153].

Small-angle X-ray scattering (SAXS), furnishes useful information on lipid phases (and domains), size, spacing, and bilayer thicknesses of liposome systems under different solution conditions [153,154,155,156]. The addition of a further detector on the same SAXS experimental station allows for performing wide-angle X-ray scattering (WAXS) experiments that provide complementary information (at the Å scale) on the hydrophobic lipid chain packing and their lamellar phase characteristics, when ordered crystalline-like phases are present in the liposome system.

Diffraction is a powerful technique for the structural investigation of lipid-based ordered (crystalline-like) systems, such as lipid multilayers, bilayers, or monolayers, and their structural organization within the liposome nanostructures [38,40]. The sharp peaks detected in the low-angle region of a diffraction pattern are connected with the (long-range) organization of the lipid nanostructures and indicate the specific crystalline lattice (and symmetry) and the unit cell repeat distance of the detected lipid phase. The signals in the high-angle region furnish indications of the short-range organization, which is connected with the lipids’ hydrocarbon chains conformation. Lipid hydrocarbon alkyl chains show a peculiar liquid-like conformation (called type α) that exhibits only a broad peak in the high-angle region of X-ray diffraction (XRD) spectra. In liposome and lipid-based nanostructures, the reference unit cell can be described by only one parameter, such as in the lamellar (1), hexagonal (2), and cubic (3-D)-phase systems. However, more complex phase diagrams can be detected when a variety of different phases coexist at the equilibrium (especially in multicomponent lipid mixtures). In any case, the crystalline structural properties of lipid bilayers and multilayers composed of different phospholipids (with saturated/unsaturated alkyl chains) exhibit similar dimensions in their unit cell (of about 5.0 × 8.0 Å^2^). In the lamellar phase, the unit cell indicates the total thickness of the lipid and water layers, while in the hexagonal phase, it corresponds to the distance between the axes of the cylinders. The diffraction technique plays a crucial role in the study of the interaction of liposomes with biological membranes, thus furnishing useful insights in important bio-processes such as physiological (or pathological) crystallizations or phase separations/transitions induced by nanoparticle interactions [37,148].

**Figure 17:**
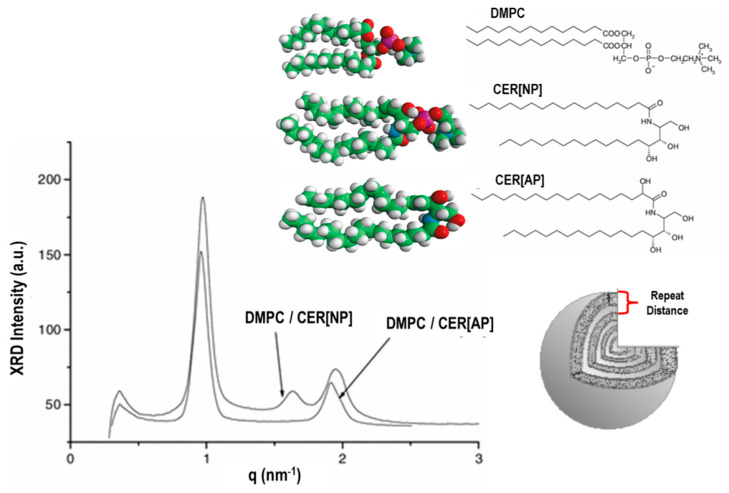
X-ray diffraction patterns for the mixed lipid system composed of DMPC/ α-hydroxy-N-stearoyl phytosphingosine CER[AP] and of DMPC/N-stearoyl phytosphingosine CER[NP] multilamellar vesicles (MLVs) at the temperature of T = 30 °C Adapted by permission from Springer Nature: [156] (Appl. Phys. A) by Kiselev et al., Copyright 2013.

A XRD study of Kiselev et al. [156] investigated the influence of the ceramide lipids on the hydration and internal nanostructure of DMPC lipid vesicles. The analysis of the XRD peaks (Figure 17) evidenced that the DMPC/CER[AP] vesicles exhibit one phase, with a membrane repeat distance of d_1_ = 6.53 ± 0.06 nm, which is larger than those observed for the pure DMPC (d_0_ = 6.27 nm) [40]. However, the DMPC/CER[NP] vesicles exhibited two phases with repeat distances of d_1_ = 6.43 ± 0.06 nm and d_2_ = 3.85 ± 0.04 nm, which correspond to the two peaks in the diffraction spectra (Figure 17). This circumstance is probably caused by the characteristic V-shaped form of CER[NP] lipids, which favors a phase separation from the DMPC lipid membrane and the formation of a new ceramide-enriched domain with a shorter periodicity (or pure CER[NP] crystal) [40].

With the use of the very intense X-ray beam available at synchrotron radiation facilities, and the dedicated instruments, it is possible to perform time-resolved experiments for the study of rapid transient processes (over millisecond time resolution and in a large range of length scales) [157,158]. More specifically, the combination of SAXS/WAXS time-resolved investigations are particularly important for the investigation of the structural properties and phase transitions in liposome nanostructures. As the lipid systems undergo complex self-assembly processes, typical of amphiphilic systems [146,159], the small-angle scattering technique allows for monitoring the structures and structural changes and the transitions of many biological amphiphilic macromolecules [152,160]. Moreover, the analysis of scattering structure factor S(q), by means of advanced theoretical approaches and computer simulation methods, allows for investigating the nanoparticles’ interactions (at the molecular level) in very different solution environments [161,162,163,164,165,166].

Small-angle neutron scattering (SANS) shares the same basic principles with SAXS. While SAXS spectra originates from the scattering of the electrons present in the materials system under investigation, and is sensitive to the hydrophilic region of a lipid bilayer, the SANS technique furnishes information on the lipids’ hydrophobic tails region, as neutrons furnish a better “contrast” for that region [151,152,153]. Moreover, neutron diffraction measurements performed on (selectively) deuterated lipids furnish an important approach for the determination of the conformation of the lipid molecules at different positions [27,40]. Therefore, SAXS and SANS represent complementary methods for a detailed description of the structure of liposomes systems.

It is worth pointing that the investigation of the structural features in lipid-based nanostructures involves the investigation of the collective behavior of a large number of interacting molecules, and helps to understand the structural and dynamic behavior of biological membranes, representing, then, a fundamental interdisciplinary topic in fields of biotechnology and pharmaceutical science [27,167].

### 8.2. Electron Microscopy and Atomic Force Microscopy (AFM) Techniques

Electron microscopy (EM) techniques allow for high-resolution visualizations of liposomes in different environments, with the possibility of resolving the details of liposomes (and nanoparticles) of varying sizes, morphologies, and lamellarities [168,169,170]. In EM, an electron beam is focused by a set of electromagnetic lenses onto the sample surface. A further set of electromagnetic lenses refocus (and magnify) the electrons scattered by the sample, thus furnishing a projected image of the sample. Sample preparation in EM techniques often requires the liposomes’ removal from their original environment, which can cause some morphological artifacts or induce structural perturbations in liposome systems [168,169,170].

In scanning electron microscopy (SEM) experiments, the (dried) sample is scanned (point by point) with an electron beam, while a detector reveals the secondary electrons that are emitted by the investigated sample. In this way, it is possible to obtain a detailed three-dimensional (3-D) picture of the nanostructures (or of the surface) present in the sample. The SEM technique also provides crucial information on the size and morphology and layered (concentric) liposomes structures [168,169,170].

In transmission electron microscopy (TEM) experiments, a small amount of sample (<100-nm thick) is placed in a vacuum chamber, while the solvent is dried prior to the microscopic analysis. This circumstance may affect the sample morphology or orientation before the analysis of their images. TEM provides the image of nanoparticles with a resolution below 1 nm in size and with enhanced contrast and image contours, thus providing important information on the surface modifications of liposomes (such as in the case of surface coating or conjugation with ligands). In TEM experiments, liposomes mostly appear as black spherical nanoparticles on a white background. TEM also offers the advantage of differentiating individual vesicles from aggregates and provides information about the lipid phase transitions, allowing for a critical and complete assessment of the liposome structural properties [169,170]. Nevertheless, TEM has several drawbacks connected with the sample pre-treatments that may cause artifacts and changes in the liposomal structure/morphology (such as vesicle swelling, shrinkage, or deformation).

To overcome many of the limitations of the TEM method, the (cryo-) transmission electron microscopy (Cryo-TEM) technique was developed [171,172]. This method allows for avoiding the liposome structure perturbation by making use of a (flash-)freezing treatment for the direct visualization of the specimen in the solid-state (without the need to eliminate the solvent). Thin liposome-hydrated films (vitrified in liquid ethane) are used prior to imaging, thus allowing for the analysis in their most native state. This rapid freezing limits the ice crystals’ formation and preserves the integrity of proteins and other biological materials. Cryo-TEM techniques provide detailed insights into lipid size, shape, internal structure (lamellarity), lipid packing, phase behavior, and drug inclusion characteristics, with resolutions in the range of 5–500 nm (defined by the film thickness). Cryo-TEM also provides high-resolution images for the investigation of complex self-assembly processes, phase changes, and dynamic processes in lipid-based nanoformulations. Moreover, the development of the cryo-tomography allows for obtaining 3D information about the structural properties of bio-materials and bio-molecules, (drug-loaded) liposomes, cubosomes, hexosomes, and other biomembrane phases [171,172].

Another important microscopic method used to analyze the liposomes’ sizes and morphologies is given by the atomic force microscopy (AFM). This technique is a type of scanning probe microscopy (SPM) that provides high resolution (of the order of fractions of a nanometer) on the 3D profile of liposome nanostructures within their native solution environment and without the need of applying vacuum [170,173]. The structural information is obtained by exploring the materials’ surface with a mechanical probe (diamond tip) which is in direct contact with the explored surface and measures the surface forces between the probe and the investigated sample (with the interatomic van der Waals forces that provide the interaction mechanism). Piezoelectric elements (sensors) facilitate precise and accurate movements of the probe. This favors the precise scanning and imaging of a large variety of surfaces, including polymers, glass, composites, and biological systems [173,174]. The AFM method can provide a 3D image of liposomes with details on size (distribution and homogeneity), shape (with a spatial resolution in the order of fractions of a nanometer), and surface modifications (due to the presence of ligands, antibodies, or polymers conjugated at the liposomes’ surface) [173,174].

### 8.3. Light (Fluorescence and Confocal) Microscopy Techniques

Light (or optical) microscopy utilizes visible light as source and a relatively simple setup of lenses, and furnishes a magnified image of the sample with a limited resolution of ~250 nm (defined by the smallest diffraction-limited spot size achievable). It furnishes useful information on the dimension, shape (homogeneity), and degree of aggregation of a liposome system or of giant unilamellar vesicles (GUVs) of hundreds of micrometers, but is unable to directly provide details on the vesicles’ lamellarity and on the structures of the SUVs [169,170]. The incorporation of low concentrations (≤1 mol%) of fluorescent probes (dyes) placed within the aqueous phase (or the lipid bilayers) permits the visualization of the liposomes’ structure (and dynamics), with a negligible impact on the structural properties of the lipid membrane [174,175]. This technique allows for the visualization of the size, shape, and fluidity of lipidic GUVs [175], while by incorporating specific probes (such as rhodamine) into the lipid bilayer allows for identifying the liposomes’ lamellarity [174], or for detecting the different conformations of lipid lateral packing (rhodamine-labeled lipids) [175].

Among the various optical imaging techniques that allow for the observation of lipid-based nanostructures, confocal laser scanning microscopy (CLSM) is a method of choice, since it facilitates the structural investigation (in an almost non-invasive way) by visualizing the liposomes’ internal structures [170]. It also allows for localizing (and quantifying) the extent of liposomes’ subcellular distribution during the drug-delivery processes [176,177,178,179]. CLSM is a crucial technique for the characterization of biomaterials and biological specimens with high-resolution images. The technique provides an image which is built by scanning one (or more) focused light beams across a slice of a sample. It gives a sharp image, as it allows for focusing on a particular focal point without interfering with the out-of-focus adjacent planes. The confocal optical system is composed of two systems of lenses: the objective lens (which views onto the image plane), and the collector lens (that acts rather as an imaging lens by using a small area point detector rather than as collectors) [174,177]. Confocal microscopy provides imaging and detection systems that are on the same focal plane (plane of interest). Recent setup upgrades allow for further processing into a 3D specimen representation by means of the volume visualization techniques. To selectively mark the view of a nanocarrier within a biological sample, a fluorescence dye (such as rhodamine 123, coumarin 6, fluorescein 5-isothiocyanate (FITC), etc.) is inserted during the sample preparation. Significant advances in the technique allow for generating high-resolution images (in a noninvasive manner) at various depths of the skin layer without the need of any sample mechanical sectioning [180].

In conclusion, no single experimental approach can combine high spatial resolution image acquisition in one instrument, while the combination of different microscopy methods allows for studying many structural features of the liposomes’ nanoformulations at different stages of the drug-delivery process [174].

**Figure 18:**
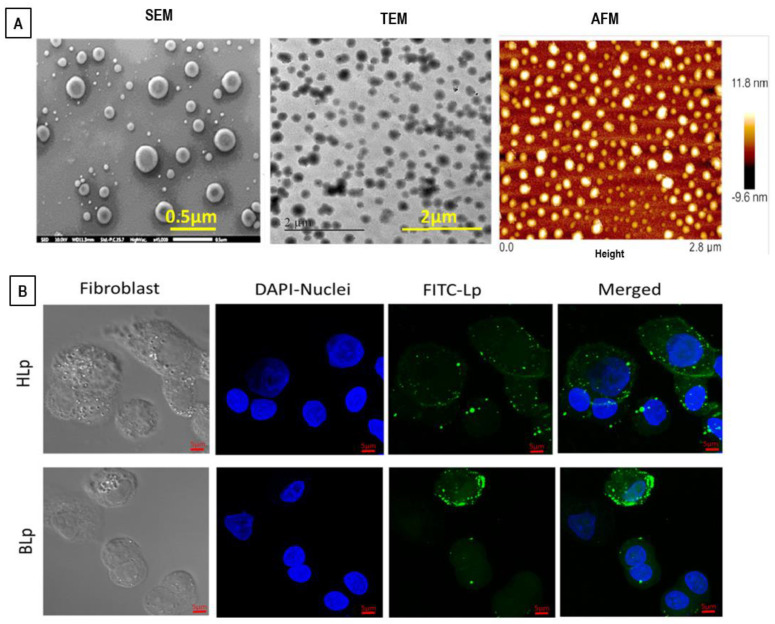
(**A**) Images of heparin-loaded liposomes (HLp), obtained by means of the SEM, TEM, and AFM techniques. (**B**) Confocal laser scanning microscopy images of cellular uptake of HLd (top) and the blank liposomes (BLp) (bottom) by fibroblast cell lines after 3 h of incubation. Blue (DAPI): nucleus; green (fluorescent HLp). Adapted with permission from Vaghasiya et al. [181], Copyright 2019 American Chemical Society.

For example, heparin-loaded liposomes (HLp) composed of L-α-phosphatidyl choline, cholesterol, and stearylamine (PC/Chol/SA, 7:3:1) have been recently prepared by the thin-film hydration method (followed by the extrusion) [181]. The nanocarrier was finally formulated as an ibuprofen-containing gel to obtain a nano-spray formulation applicable for topical drug-delivery treatments (HLp-Ibu-NSG). The blank liposome (BLp) had a mean diameter of (181 ± 23), polydispersity index (PDI) of (0.278 ± 0.093), and zeta potential of (+41.6 ± 3.98) mV, while upon the encapsulation of heparin, the HLp exhibited a slight increase in the size (204 ± 12 nm) and PDI (0.41 ± 0.072), and a slight decrease in the zeta potential of (+38.3 ± 4.3) mV. Those results obtained by the dynamic light scattering were confirmed by means of the SEM, TEM, and AFM techniques (Figure 18A), which evidenced an average liposome diameter of around d = 200 nm. Moreover, fluorescently labelled HLp were prepared for cellular uptake studies. The accumulation ability of cationic HLp in mouse fibroblast cells was investigated by using confocal laser scanning microscopy (Figure 18B). Fluorescent confocal micrographs showed that fibroblasts exposed to the HLp (green fluorescence) for 3 h accumulated liposomes (in significant numbers) inside the cytoplasm of the cells (Figure 18B). No significant difference in cellular accumulation was observed between HLp and BLp [181].

HLp exhibited significant healing of a wound in vitro (scratch assay, fibroblast cells) and in vivo (wound healing in Sprague–Dawley rats) at a low dose. In the rat model of frostbite injury, the HLp-Ibu-NSG formulation demonstrated significant reductions in the wound area (up to ~96%), and improvements of histopathology in 14 days, as compared to the control groups, while no edema or erythema were detected (in the affected area) during the post-treatment of HLp-Ibu-NSG [181].

### 8.4. Dynamic Light Scattering Technique

Dynamic light scattering (DLS) furnishes important information on the structure and dynamic properties of nanoparticles in solution, such as bio-macromolecules, colloids, nanoemulsions, liposomes, and gels [182,183]. In a DLS experiment, the fluctuations in the scattered light intensity is connected with the scattered electric field correlation function, which yields information on the nanoparticles’ Brownian motion through the Stokes–Einstein relation D = k_B_T/6πρR_H_, which expresses the (translational) diffusion coefficient D as a function of the particles’ hydrodynamic radius R_H_, the absolute temperature T, and the solvent viscosity ρ (where k_B_ is the Boltzmann constant) [182,183]. Analysis of the scattering intensity also allows for obtaining the liposomes’ nanoparticles size distribution [152,183].

### 8.5. Zeta (ζ) Potential Technique

**Figure 19:**
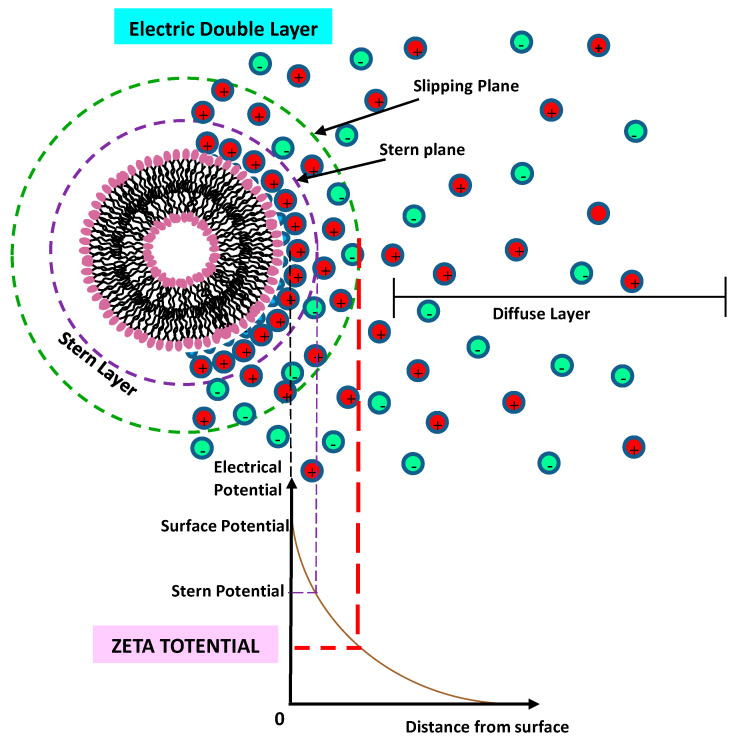
Diagram indicating the electrical potential as a function of the ionic concentration (and distance from the charged surface) of a liposome suspended in a dispersion medium.

The zeta (ζ) potential measurement represent a useful tool for addressing the electrostatic effects in charged nanocarriers [184]. It is regulated by the nature and distribution of the surface charge of liposomes and depends on the lipid composition and their headgroup charges. The zeta potential is an important factor that strongly regulates the liposomes’ colloidal stability, their biodistribution, pharmacokinetics, cellular affinity, and drug-internalization processes. A liposome nanocarrier dispersed in an aqueous solution acquires a surface charge caused by the ionization of surface end-groups (or the adsorption of charged species on its surface) (Figure 19). The dispersed counter-ions will surround the surface of the particle (Stern layer), while the zeta potential represents the electrical potential at the nanoparticle interface. The zeta potential is a key indicator of the colloidal stability of a liposome’s dispersions, as it expresses the degree of electrostatic repulsion between nanoparticles in a dispersion. Nanoparticles with high (negative or positive) zeta potentials are electrically stabilized (and have high colloidal stability), while the nanoparticles that exhibit low zeta potentials tend to aggregate or flocculate.

The zeta potential ζ of a colloidal nanoparticle is obtained by measuring the electrophoretic mobility μE using principles of phase analysis light scattering (PALS) [184,185]. It is worth noticing that a suitable combination of light-scattering and zeta-potential techniques allows for a detailed investigation of electrostatic interactions in charged liposomes, and furnishes useful information on the interactions involved between complex charged liposome nanocarriers and target cells or tissues [186,187,188,189].

### 8.6. Other Complementary Characterization Techniques

Detailed information on the liposome population over a wide range of sizes can be determined by means of the size-exclusion chromatography (SEC) technique [190]. SEC and high-performance size exclusion chromatography (HPSEC) allows for physically separating (and quantifying) liposomes from small solutes, or for subdividing different liposome subpopulations. Those techniques allow for the determination of the size (distribution and polydispersity), stability, bilayer permeabilization, as well as liposome formation and reconstitution processes. SEC also represents a powerful method for the study of the encapsulation/interaction of a large variety of compounds, including small solutes (such as drugs, surfactants, etc.) or large macromolecules (such as peptides, proteins, and nucleic acids) in liposomes [190]. Alternatively, the field-flow fractionation (FFF) separation techniques offer a physical separation method of complex and inhomogeneous liposomes samples, which potentially cannot be characterized by other separation methods, such as SEC. The absence of a stationary phase in the FFF technique causes a reduced interaction with surfaces or column packing materials [191,192].

Many other complementary techniques can be employed for the determination of the affinity of active drugs (and macromolecules) for liposomes’ inclusion, including various spectroscopy techniques such as ultraviolet (UV), FT-infrared (FT-IR), electron paramagnetic resonance (EPR), electron spin resonance (ESR), and circular dichroism [191,192,193]. Furthermore, complementary biophysical techniques, such as the differential scanning calorimetry (DSC) [193] and the isothermal titration calorimetry (ITC) [194], allow for the study of the thermodynamics of drug–lipid interactions (and the associated binding processes). Moreover, nuclear magnetic resonance (NMR) is a quantitative spectroscopy technique for the (non-destructive and non-invasive) study of the drug–liposomes interactions, and furnishes crucial information on changes in the bilayer structure or dynamics of lipid molecules and lipid-based nanostructures (at a sub-Ångstrom resolution) [195,196].

In conclusion, the use of high-performance characterization techniques, combined with advanced structural modeling, have an important impact on the quality control in lipid nanocarriers’ post-formation processes.

## 9. Technology Transfer and Regulatory Perspectives

The assessment of the quality of pharmaceutical nanoformulations and health care products is a mandatory step before their technology transfer and their introduction into the market. Although a growing number of liposome systems are able to prove their beneficial action in preclinical trials, only liposome nanoformulations that are efficient in clinical trials will enter into the clinic. In the last decades, a wide range of liposome formulations were successfully translated into the clinic, while others are in different phases of their clinical investigation [17,18]. The quality of pharmaceutical products is intimately connected with their performance, stability, and absence of contamination, and it is certified through their efficacy in obtaining the therapeutic benefits indicated on the products’ labels [197]. As many liposome nanoformulations involve the use of novel drugs and complex interactions between the various molecular components, a set of physicochemical and biopharmaceutical characterization methods are required before liposomes nanoformulations are recognized (and marketed) as biomedical products [198]. The high costs of production and the stringent requirements for assessing their benefits (or risks) can represent a limit to an efficient technology transfer. The specific requirements of a liposome nanoformulation depend on the pharmaceutical type of intervention, the target patient population, and the corresponding route of administration. In this respect, the regulatory agencies outline the number of requirements needed in terms the quality, safety, and effectiveness of the liposome-based products [197,199].

In the last decades, the European and US regulatory agencies have promoted guidelines to push manufacturers to perform accurate pre-authorization investigations for assessing the quality, safety, and efficacy profile of the new proposed pharmaceutical products. Those regulatory agencies publish periodic scientific guidelines on human medicines that are harmonized by the International Council for Harmonisation of Technical Requirements for Registration of Pharmaceuticals for Human Use (ICH) [200,201,202]. Concerning the new liposome nanoformulations, useful guidelines can be found in the reflection paper released by the European Medicine Agency (EMA) (for intravenous liposomal products) [201] and the draft guidance of the Food and Drug Administration (FDA) (for liposome drug products) [202]. The information provided by those agencies helps to define (harmonized) quality standards concerning:

- the assessment of the benefit/risk of the liposomal systems;
- definition of the critical quality attributes of the final product;
- guide for the formulation studies in the early stages of development (designing preclinical/clinical trials);
- support to the pharmaceutical quality control system and sustaining the post-marketing variations.

In particular, those agencies require a detailed description of the liposomes’ composition (quality/purity of lipids used), a characterization of their physicochemical properties, manufacturing process (and controls), and stability in physiological environments, a characterization of their functionality (related to the specific application), and clinical pharmacology studies (pharmacokinetics, bioavailability, bioequivalence tests).

Concerning the future perspectives for an efficient technology transfer and regulatory activities, a synergistic action of joint research centers may contribute to stimulate exploratory research for the translation to the clinic of liposome products and nanomedicines. More specifically, the new discoveries could stimulate the identification of the main regulatory issues and the corresponding design strategies, in collaboration with the world regulation authorities. In this respect, the creation of a regulatory consortium, by including a wide community of laboratories, industries, stakeholders in regulation, and end users, could help establish a permanent forum for the efficient transfer of the new scientific results into regulatory strategies, and to update the regulatory issues (including their harmonization). The network between scientists, regulation agencies, and product developers can stimulate the advancement of regulatory issues in the field of pharmaceutics and nanomedicine, through the identification of the safety issues induced by nanomedicines, the development (and validation) of new standards for analytical (or experimental) methods, and assays requested by the regulators.

### The Quality by Design (QbD) Method

Recently, a Quality by Design (QbD) approach has been proposed to improve the quality of pharmaceutical products [203,204]. QbD is a new perspective based on the analysis and study of potential factors that influence the quality of the pharmaceutical products through a risk-assessment process. The application of the QbD strategy in the pharmaceutical developments helps to influence the quality, the achievements, and the success of the pharmaceutical nanoformulation products. For this reason, the QbD approach has been recommended by drug regulatory agencies such as the FDA and EMA for the development and manufacturing of better-quality drug formulations.

A QbD approach includes the following crucial stages, as follows [203,204]:

(1) A definition of the quality target product profile (QTPP), based on some specific properties of the product, in order to ensure the desired quality, safety, and efficacy of the drug product, and by considering the critical factors of the administration (such as specific route, dosage, bioavailability, strength, and stability);
(2) The identification of the main factors, such as the critical quality attributes (CQAs) of the targeted product, the critical material attributes (CMAs), and the critical process parameters (CPPs), which are related to the selected production method;
(3) The identification of the risk assessment (RA) through a science-based process that ranks the parameters’ impact on the CQAs of the product, and identifies (between them) the critical material attributes (CMAs) and critical process parameters (CPPs);
(4) The development of a design space (DS), i.e., a study of the combination and interaction (in a mathematical form) of the input variables (such as the CMAs and CPPs) and their impact on CQAs and the process parameters (to ensure desired product quality). To establish the design space with a minimum number of experiments, the Design of Experiments (DoE) strategy is used;
(5) The definition (and implementation) of a control strategy (CS), with the aim of stimulating the continuous products’ improvement;
(6) A life cycle management.

In recent years, a growing number of investigations have used the QbD approach to facilitate the identification of the main risk factors that may impact liposome nanoformulation formation processes, in order to improve the quality of product, through the analysis and the control of the formulation’s materials and manufacturing variables [204,205,206].

## 10. Conclusions

In this article, we analyzed the main features of the formation techniques of liposome nanocarriers with a special focus on the structural parameters and the critical factors that influence the development of a suitable and stable nanoformulation. Traditional methods and novel approaches for liposome preparation are discussed, with the objective of updating the reader on recent developments and providing future directions for research and development. Conventional techniques for liposome preparation still remain very popular, as they are simple to implement and do not require sophisticated equipment for their instrumental upgrade. However, these techniques may not be suitable for the processing of several (bio-) molecules, as they may produce structural (and then functional) alterations. With the recent progress in nanotechnology and biomedicine, novel manufacturing methods for the facile encapsulation of both hydrophobic and hydrophilic molecules (with no need for organic solvents and/or sophisticated equipment) are highly desired. Moreover, the critical issue of system instability for liposomes loaded with hydrophilic materials requires new strategies that will achieve acceptable loading while aiming at targeting cargo delivery at the site of interest. Furthermore, the difficulties of achieving an easily scalable process for mass production, and of achieving elevated encapsulation efficiencies, still represent the further drawbacks of conventional liposome formation processes. In this respect, the study of novel formation approaches, together with the introduction of new technologies, may stimulate the production of advanced liposome nanoformulations from the laboratory, which will be suitable for industrial-scale production and even more effective clinical applications.

## Figures and Tables

**Table 1:** Summary of the main features obtained by using different formation methods.

Method	Advantages	Disadvantages	Liposomes	Ref.
Thin-Film Hydration (Bangham method)	Simple procedure, good encapsulation efficiency (EE) (both with small and large drugs)	Large use of organic solvent (difficult to remove) lower EE for water-soluble drugs, small-scale production, no particle size control, time consuming, sterilization needed	Polydisperse MLVs	[66,67,68]
Detergent Removal (Depletion) Method	Simple procedure, good EE (both with small and large drugs)	Need of large amount of organic (and residual) solvent, poor EE (for lipophilic drugs), low final liposome concentrations (low yield), time consuming, sterilization issue	MLVs, LUVs	[71,72,73,74,75,76]
Solvent (Ethanol/Ether) Injection	Simple, rapid, reproducible Ether injection gives greater EE.	Removal of ethanol is difficult, as it forms azeotrope with water, difficult to handle biologically active macromolecules in ethanol, possible nozzle blockage (ether system), sterilization issue	SMVs, SUVs	[81,82,83,84,85]
Reverse-Phase Evaporation	Simple process, suitable EE	Large quantity of organic solvent, not suitable for fragile (bio-) drugs, time consuming, sterilization issue	MLVs, LUVs	[88,89,90]
**Novel Technologies**				
Freeze Drying (Lyophilization)	Low organic solvent residue, suitable for large-scale production, prevents the physical degradation of liposomes during storage, increases liposomes’ shelf-life	Time- and energy-consuming, may induce (structural/size) alterations in formed vesicle, loss of encapsulated material, sterilization issue	MLVs, LUVs SMVs, SUVs	[100,101,102,103,104,105]
SC Reverse-Phase Evaporation (SC-RPE)	Control of particle size, possible in situ sterilization, low organic solvent (env. friendly), quickly encapsulates both hydrophilic and lipophilic materials, large-scale production	high pressure used, high capital cost, low encapsulation efficiency, low liposome stability	LUVs, MLVs	[109,110,111,112]
Supercritical Anti-Solvent (SAS)	Relatively simple (and repeatable), control of particle size, low organic solvent (and residues), in situ sterilization,	High capital cost, low yield and EE, possible aggregation of particles, presence of residual (toxic) solvents in the final product	LUVs, MLVs	[113,114]
Rapid Expansion of a Supercritical Solution (RESS)	Control of particle size, possible in situ sterilization, low organic solvent consumption (that can be reused)	low yield and EE, high pressure (up to 250 bar) used, high production cost, poor solubility of (polymer-based) biomaterials in SC-CO_2_, difficulty of the separation between co-solvents and vesicles during the depressurization process, may involve nozzle blockages	OLMs, MLVs, ULVs	[115,116,117,118,119,120]
Supercritical Assisted Liposome Formation (SuperLip)	Continuous and replicable process, encapsulates hydrophilic drugs, high EE, low solvent residue	time-consuming process, requires high pressure, high capital cost	LUVs, MLVs	[121,123]
Depressurization of an Expanded Liquid Organic Solution (DELOS)	Simple and rapid process, control of the liposomes size, possibility to obtain small sizes, shape uniformity/homogeneity, and good stability, possibility to reduce sterols use, high EE (hydrophilic drugs)	residual organic solvent, nozzle blockage	LUVs, MLVs	[124,125,126,127]
Microfluidic (Micro Hydrodynamic Focusing—MHF), (Microfluidic Droplets—MD), (Pulsed Jet Flow—PJF)	Good particle size control, possibility to upgrade to novel methods for liposome preparation (lab on chip)	organic solvent (difficult to remove), not suitable for bulk production, high cost of microfluidic channels	SUVs, LUVs (for MHF), GUVs (MD), GUVs (PJF)	[128,129,130,131,132,133,134,135]
Membrane Contactor	Fast process, high EE, good size (distribution) control, scaling-up abilities (for industry)	possibility of clogging the pores, membrane blockage, high temperature, sterilization issues	MLVs,	[136,137,138]

## Data Availability

Not applicable.
